# Rumen unprotected glucose tends to promote intramuscular fat deposition via microbial volatile fatty acid-mediated metabolic reprogramming in cattle: a multi-omics perspective through the rumen-jejunum axis

**DOI:** 10.1186/s40104-026-01419-6

**Published:** 2026-05-25

**Authors:** Yang Song, Ning Liu, Zan Liang, Zuo Wang, Tingting Chu, Qianglin Liu, Weijun Shen, Xiangmin Yan, Fachun Wan

**Affiliations:** 1 College of Animal Science and Technology, Hunan Agricultural University, Changsha, 410128 China; 2 Yuelushan Laboratory, Changsha, 410128 China; 3 Institute of Animal Husbandry, Xinjiang Academy of Animal Husbandry Sciences, Urumqi, 830000 China

**Keywords:** Lipid deposition, Rumen-jejunum axis, Rumen-unprotected glucose, Rumen-protected glucose, Volatile fatty acids

## Abstract

**Background:**

The regulatory effects of glucose absorption at different sites (rumen vs. small intestine) on lipid metabolism exhibit significant variation in beef nutrition. This study aimed to investigate the regulatory pathways of rumen-protected or unprotected glucose on lipid metabolism through the rumen-jejunum axis in Xinjiang Brown cattle.

**Results:**

Thirty Xinjiang Brown cattle (females) with similar initial weight (410 ± 22.4 kg) were randomly assigned to 3 treatment groups (*n* = 10 animals per group). The experimental groups were fed a basal diet with the following daily supplements per head: 150 g palmitate coating (CON group), 150 g palmitate coating plus 150 g rumen-unprotected glucose (RUG group), and 300 g rumen-protected glucose (containing 50% glucose; RPG group). The experiment lasted for 70 d. Supplementation with both rumen-unprotected glucose and rumen-protected glucose increased chest width (*P* = 0.001), chest girth (*P* = 0.013), abdominal girth (*P* = 0.002), backfat thickness (*P* = 0.041), omental fat weight (*P* = 0.047), as well as serum concentrations of insulin (*P* < 0.001), glucagon-like peptide-1 (*P* < 0.001), and 5-hydroxytryptamine (*P* < 0.001), while decreasing the content of deoxycholic acid (*P* < 0.001) in the jejunum. The inclusion of rumen-unprotected glucose resulted in a trend toward higher intramuscular fat (IMF) (*P* = 0.064) in the longissimus thoracis, along with significant increases in C14:0 (*P* = 0.042), C15:0 (*P* = 0.014), and marbling score (*P* = 0.048), as well as significant reductions in drip loss (*P* = 0.022) and shear force (*P* = 0.041). These changes were accompanied by significant increases in dry matter intake (*P* = 0.001), ruminal concentrations of acetate (*P* = 0.022) and propionate (*P* = 0.011). The rumen-protected glucose supplementation elevated serum glucose (*P* < 0.001) level, while enhancing digestibility of ether extract (*P* = 0.027) and neutral detergent fiber (*P* = 0.027). Neither rumen-unprotected glucose nor rumen-protected glucose affected the alpha diversity and beta diversity of ruminal and jejunal microbiota (*P* > 0.05), but the differential bacterial biomarkers were either positively or negatively correlated with chest girth, abdominal girth, marbling score, backfat thickness, and deoxycholic acid (*P* < 0.05).

**Conclusion:**

Rumen-unprotected glucose supplementation enhances IMF deposition and meat quality attributes through microbial volatile fatty acid-driven metabolic reprogramming.

**Supplementary Information:**

The online version contains supplementary material available at 10.1186/s40104-026-01419-6.

## Background

In the global beef cattle industry, optimizing fat deposition in fattening cattle, particularly the formation of intramuscular marbling, represents a critical objective for enhancing beef quality and economic value [1, 2]. Glucose serves as a pivotal nutrient in bovine metabolism, playing a central role in regulating intramuscular adipose tissue deposition and beef marbling quality [3]. In adipocytes, glucose-derived carbons contribute over 70% of acetyl-CoA for de novo lipogenesis, with restricted lipogenic capacity rendering adipocytes entirely dependent on glucose as the primary carbon source for fatty acid synthesis [4]. Strategic enhancement of glucose availability has been shown to promote marbling development, as evidenced by improved marbling scores in early-weaned calves and cattle fed corn-based diets that optimize glucose supply for intramuscular adipocytes [5, 6]. Notably, the metabolic pathways of glucose in the diet are fundamentally different due to the different absorption sites: if it is absorbed in the rumen, it will be rapidly degraded by microbial fermentation into short-chain fatty acids (SCFAs), whereas intestinal absorption of rumen protected glucose (RPG) enables direct entry into portal circulation, bypassing ruminal catabolism [7]. This spatial dichotomy in glucose utilization establishes distinct regulatory paradigms for lipid metabolism, with rumen-derived SCFAs and intestinally absorbed glucose potentially activating divergent signaling pathways to modulate systemic energy partitioning.

The "rumen-jejunum axis" emerges as a critical interface governing these nutrient-host interactions. Spatial heterogeneity in microbial ecosystems and metabolic environments along the gastrointestinal tract creates compartment-specific regulatory nodes [8, 9]. While the rumen dominates carbohydrate fermentation, producing SCFAs at magnitudes exceeding intestinal output by orders of magnitude [10], post-ruminal segments contribute to nutrient fine-tuning through bile acid metabolism, enteroendocrine signaling, and hepatic feedback mechanisms [11]. Microbial metabolites and nutrient flows along this axis exhibit bidirectional cross-talk, as demonstrated by ruminal interventions altering pH in the jejunum, ileum, and cecum fermentation patterns [12, 13]. Short-chain fatty acids, particularly acetate, propionate, and butyrate, function as key nodal molecules in this axis, not only as energy substrates but also as signaling mediators that regulate GLP-1 secretion, bile acid cycling, and hepatic gluconeogenesis [14]. However, in ruminants where SCFA production overwhelmingly originates from the rumen, their role as inter-organ messengers between the rumen and distal gut remains ambiguous compared to non-ruminant gut-liver-brain axis models [15]. We hypothesize that dietary supplementation with RPG or rumen unprotected glucose (RUG) induces divergent lipid metabolic reprogramming in Xinjiang Brown cattle, mediated through distinct regulatory pathways. This study aims to elucidate the differential regulatory mechanisms of dietary RUG (rumen fermentation) versus RPG (intestine-absorbable) on lipid reprogramming, based on the "rumen-jejunum axis" framework in Xinjiang Brown cattle.

## Materials and methods

### Animal ethics statement

All experimental protocols were approved by the Animal Ethics Committee of Hunan Agricultural University (Approval No. 20250126) and strictly complied with relevant ethical guidelines and regulations.

### Animal experimental design

The animal trial was conducted from March to June 2025 at Tohulasu Animal Husbandry Co., Ltd. (Yining City, Xinjiang, China). Thirty healthy Xinjiang Brown cattle (females; average body weight: 410 ± 22.4 kg) were randomly assigned to three groups (*n* = 10 per group) using a completely randomized design: (1) CON group: basal diet +150 g/head/d palmitate coating; (2) RUG group: basal diet + 150 g/head/d palmitate coating + 150 g/head/d RUG; and (3) RPG group: basal diet + 300 g/head/d RPG (the additive amounts of RUG and RPG were determined based on previous studies [16, 17]). The trial included a 15-day adaptation period followed by a 70-day formal experimental period. The animals were fed twice daily on an ad libitum basis, with the feed ration adjusted every 30 d, which resulted in a dry matter intake (DMI) maintained at 2%–2.5% of body weight. Glucose additives were administered by thoroughly mixing pre-weighed RUG (feed-grade; purity ≥ 99%) or RPG (purity = 50%; rumen bypass rate ≥ 85%; provided by Hunan Pufeike Biotechnology Co., Ltd., Changsha, China) with a small portion of the total mixed ration (TMR) in individual feeding troughs before distributing the remaining TMR. Cattle were housed in a barn system with free access to water (each cattle was assigned to an independent stall). Cattle were fed a TMR twice daily (06:00 and 16:00), with daily feed residuals maintained at ≥ 5% of offered feed and measured at 11:00 and 21:00. The basal TMR (concentrate-to-forage ratio for 35:65) was formulated to meet or exceed nutrient requirements for beef cattle (NRC, 2015; Table 1) [18].

**Table 1:** Ingredients and chemical composition of diets (DM basis), g/kg

Items	Treatment		
	CON	RUG	RPG
Ingredient^1^			
Alfalfa	131.8	131.8	131.8
Corn straw	85.6	85.6	85.6
Cotton shell	122.5	122.5	122.5
Whole plant corn silage	128.0	128.0	128.0
Distillers grains	158.6	158.6	158.6
Corn	201.7	201.7	201.7
Cottonseed meal	59.8	59.8	59.8
Extruded cottonseed	11.2	11.2	11.2
Soybean meal	16.4	16.4	16.4
Flaxseed meal	41.1	41.1	41.1
Wheat bran	14.9	14.9	14.9
Na_2_HCO_3_	7.5	7.5	7.5
NaCl	5.6	5.6	5.6
Premix^2^	14.9	14.9	14.9
Yeast powder	0.4	0.4	0.4
Rumen-unprotected glucose, g/head/d	—	150.0	—
Rumen-protected glucose (effective content 50%), g/head/d	—	—	300.0
Palmitate coating, g/head/d	150.0	150.0	—
Chemical composition			
DM	589.7	589.7	589.7
OM	519.1	519.1	519.1
GE, MJ/kg	16.87	17.1	17.1
CP	138.9	138.9	138.9
EE	32.5	32.5	32.5
NDF	526.4	526.4	526.4
ADF	256.0	256.0	256.0

*GE* Gross energy, *DM* Dry matter, *CP* Crude protein, *OM* Organic matter, *EE* Ether extract, *NDF* Neutral detergent fiber, *ADF* Acid detergent fiber

^1^ All values were the determined averages from the monthly total mixed ration analyses

^2^ Per 1 kg of premix contained 12,000 IU of vitamin A, 3,500 IU of vitamin D_3_, 40 mg of vitamin E, 3,139 mg of Mn, 4,550 mg of Zn, 1,729 mg of Cu, 1.82 mg of I, 2,197 mg of Fe, 0.62 mg of Se, and 0.04 mg of Co

### Feed and feces sample collection and chemical analysis

On d 0 and 71 of the experiment, all cattle were fasted (with free access to water) for 12 h prior to measurement of body weight (BW) and body size indices.. The average daily gain (ADG) was calculated as: ADG = (Final BW − Initial BW)/70. Throughout the experimental period, the feed intake and refusals (orts) for each animal were recorded daily and used to calculate dry matter intake (DMI). Feed conversion ratio (FCR) was calculated as DMI divided by ADG. A 5-day digestibility trial (d 65–69) was conducted to collect TMR and fecal samples. Fecal samples (approximately 300 g) of all cattle were collected rectally at 8:00 and 15:00 from d 65 to 69. A subsample (100 g) was homogenized with 10 mL of 10% tartaric acid to stabilize nitrogen content. Fresh basal diets and orts were collected daily then pooled per cattle for subsequent subsampling. All samples (feed, orts, feces) were stored at −20 °C. Apparent nutrient digestibility was determined using the endogenous indicator method (acid-insoluble ash; AIA). The feed and feces samples were oven-dried at 65 °C for 48 h and ground to pass through a 0.9-mm screen before chemical composition analysis. According to AOAC (2005) [19], samples were analyzed in duplicate for: dry matter (DM; method 934.01), crude protein (CP; method 954.01), organic matter (OM; method 942.05), and ether extract (EE; method 920.39). The neutral detergent fiber (NDF) and acid detergent fiber (ADF) levels were determined using the method of Van Soest et al. [20]. Nutrient digestibility was calculated using AIA as described by Van Keulen and Young [21]:

$$\text{Digestibility }\left(\%\right)=100-\left(\frac{A_1}{A}\times \frac{B}{B_1}\right)\times 100$$

where *A* is the content of a given nutrient in the diet (%), *A*_1_ is the content of the same nutrient in the feces (%), *B* is the content of AIA in the diet (%), and *B*_1_ is the content of AIA in the feces (%).

### Blood sample collection and chemical analysis

Blood samples were collected before morning feeding using 5-mL plain serum vacuum tubes (Aosite Bioengineering Co., Ltd., Heze, China). The samples were centrifuged at 4 °C and 3,000 × *g* for 15 min, and the harvested serum was stored at −80 °C for subsequent biochemical and hormonal analyses. Serum biochemical parameters, glucose (GLU), total cholesterol (CHO), and triglycerides (TG) were quantified using a fully automated biochemical analyzer (MINDRAY BS-360E, China). Serum hormone profiles were quantified using commercially available enzyme-linked immunosorbent assay (ELISA) kits. Insulin (INS; Kit No. F4035-B), glucagon-like peptide-1 (GLP-1; Kit No. F3964-B), cortisol (Kit No. F3991-B), epinephrine (EPI; Kit No. F3976-B), triiodothyronine (T3; Kit No. F6751-B), thyroxine (T4; Kit No. F6752-B), leptin (LEP; Kit No. F3978-A), adiponectin (ADP; Kit No. F6768-A), and glucagon (Kit No. F6748-B) were analyzed using kits from Shanghai Kexing Trading Co., Ltd. (China). 5-Hydroxytryptamine (5-HT; Kit No. YT-138102) was analyzed using a kit from Jiangsu Enzyme Immuno Industry Co., Ltd. (China). Absorbance (OD values) was measured at 450 nm with a microplate reader (Rayto RT-6100, China), and hormone concentrations were calculated based on standard curves.

### Slaughter performance and meat quality analysis

At the conclusion of the trial, these cattle were slaughtered at a commercial slaughterhouse following the commercial slaughter procedure. Slaughter performance parameters, including live weight at slaughter, hot carcass weight (used to calculate dressing percentage as hot carcass weight divided by live weight multiplied by 100%), backfat thickness, liver weight, omental fat weight, mesenteric fat weight, perirenal fat weight, loin eye area (longissimus thoracis, abbreviated as LT), marbling score, LT pH and LT color were recorded. Tissue weights were measured using an electronic balance. The carcass was longitudinally split, and backfat thickness between the 12^th^ and 13^th^ ribs on the right carcass side was measured using a vernier caliper (Starrett^®^, Athol, Massachusetts, USA) [22]. Marbling was assessed using the Japanese Beef Marbling Standard (BMS), which grades meat on a scale from A1 (poor) to A5 (excellent). For statistical analysis, BMS grades (A1 to A5) were converted to numerical scores of 1 to 5, respectively [22]. The rib eye area was traced onto acetate paper and quantified using ImageJ 1.54p (National Institutes of Health, Bethesda, MD, USA). Meat color parameters (lightness, L*; redness, a*; yellowness, b*) of the LT muscle were measured using a CR-410 chroma meter (Konica Minolta, Tokyo, Japan) in the CIELAB color space [22]. Longissimus thoracis muscle pH was determined at 45 min and 24 h postmortem using a portable pH meter (Testo 205, Testo AG, Schwarzwald, Germany) [22]. For meat color, pH, and backfat thickness measurements, triplicate readings were averaged (*n* = 10; the mean of three measurements).

Within 45 min postmortem, LT muscle samples from the 12^th^–13^th^ rib region were collected, divided into two portions: one stored at −80 °C for fatty acid profiling and the other at −20 °C for meat quality analyses. For fatty acid composition, freeze-dried LT muscle powder was weighed in duplicate, and fatty acid methyl esters (FAMEs) were prepared via direct methylation. FAME profiles were analyzed using an HP-88 capillary column (100 m × 0.25 mm × 0.20 μm; Agilent 8860 C, Agilent Technologies, USA) with high-purity nitrogen as the carrier gas (1 mL/min). Injection volume was 1 μL, and the detector temperature was maintained at 250 °C. The oven temperature program was: initial 100 °C (hold 13 min), ramp to 180 °C at 10 °C/min (hold 6 min), then to 200 °C at 1 °C/min, and finally to 240 °C at 20 °C/min [22]. Fatty acid methyl esters were identified by retention time comparisons with certified standards (Tanmo Technology Co., Ltd., Changzhou, China) and expressed as weight percentages of total FAMEs. Drip loss was measured by suspending a meat sample (approximately 6 cm ×3 cm ×3 cm and 50 g) in plastic bottles (*n* = 10) and placing them in a refrigerator at 4 °C for 24 h [23]. The surface water was removed with filter paper. Drip loss was calculated as the difference in weight of the steak before and after suspension, expressed as a percentage of the initial weight. Cooking loss was measured by placing the steaks (approximately 2.5 cm thick) in a resealable pack bag and immersing them in a water bath at 80 °C until their internal temperature reached 70 °C, monitored using a digital thermometer (HI 9041, Hanna Instruments Ltd., Bedfordshire, UK). The surface water was removed with filter paper after cooling to room temperature. Cooking loss was calculated as the difference in weight of the steak before and after cooking, expressed as a percentage of the initial weight [24]. The cooked steaks were cooled at 4 °C for 24 h prior to determining Warner–Bratzler shear force (WBSF). Eight cuboids (2 cm ×1 cm ×1 cm) were removed from the cooked steak parallel to the fiber orientation and then sheared using a Warner–Bratzler shear force machine (G-R Manufacturing, Manhattan, KS, USA) with a V-shaped Warner–Bratzler cutting blade (speed; 60 mm/min; 1,000 Newton (N) load cell) [24]. The maximum shear force was recorded during this process. For the proximate analysis, all external fat and connective tissue were removed from the lyophilized LT muscle. Moisture, protein and fat content of the LT muscle were determined in triplicate, following the guidelines outlined by the Association of Official Analytical Chemists (AOAC, 2005). Muscle nitrogen was analyzed utilizing the Dumas combustion method (D60; Hanon, Shandong, China), and protein was calculated using a 6.25 nitrogen-to-protein conversion factor [22]. Fat content was determined using a Soxhlet apparatus with petroleum ether as the extraction solvent [22].

### Rumen fluid and jejunal chyme collection and analysis

All animals were slaughtered 4 h after morning feeding (with free access to water), and rumen and jejunal digesta samples were collected immediately after slaughter. Rumen fluid were collected from rumen abdominal sac, and jejunal chyme were taken from a site 3 m distal to the Treitz ligament (proximal jejunum). Rumen fluid was collected and filtered through four-layer sterile cheesecloth, after which jejunal chyme and 5 mL aliquots of the filtered rumen fluid were transferred to cryovials, flash-frozen in liquid nitrogen, and stored at −80 °C for subsequent analysis of fermentation parameters including pH, volatile fatty acid (VFA), ammonia nitrogen (NH_3_-N), microbial crude protein (MCP), bacterial community composition, and metabolomic profiles. The pH was measured in triplicate using a portable pH meter (LE438-2 M, Mettler Toledo), and the mean value was recorded. VFA concentrations were quantified using an Agilent 7890B gas chromatography (Agilent Technologies, Santa Clara, CA, USA) equipped with a polar capillary column (AT-FFAP: 30 m × 0.32 mm i.d. × 0.5 μm film thickness) following the procedures described by Wang et al. [25]. Ruminal NH_3_-N concentration was measured spectrophotometrically (SpectraMax M5; Molecular Devices, San Jose, CA, USA) at an absorbance of 630 nm according to Liu et al. [26]. Ruminal MCP concentration was determined using the bicinchoninic acid (BCA) method (Cat No. MA0082-2; Meilun biotechnology, Dalian, China) on a spectrometer (SpectraMax M5 platform, Molecular Devices, San Jose, CA, USA) according to Makkar et al. [27]. The secondary bile acids in the jejunum, including deoxycholic acid (DCA; Kit No. YT0161-VA) and lithocholic acid (LCA; Kit No. YT14771-A), were quantified using commercially available ELISA kits (Jiangsu Enzyme Immuno Industry Co., Ltd., China). Absorbance (OD values) was measured at 450 nm with a microplate reader (Rayto RT-6100, China), and concentrations were determined by interpolation from standard calibration curves.

Total microbial genomic DNA was extracted from samples using the E.Z.N.A.^®^ DNA Kit (Omega Bio-tek, Norcross, GA, USA) according to manufacturer’s instructions. The quality and concentration of DNA were determined by 1.0% agarose gel electrophoresis and a NanoDrop2000 spectrophotometer (Thermo Scientific, USA). For bacterial community, the bacterial 16S rRNA genes were amplified using the universal bacterial primers 27 F (5′-AGRGTTYGATYMTGGCTCAG-3′) and 1492R (5′-RGYTACCTTGTTACGACTT-3′). Primers were tailed with PacBio barcode sequences to distinguish each sample. Amplification reactions (20-μL volume) consisted of 5 × FastPfu buffer 4 μL, 2.5 mmol/L dNTPs 2 μL, forward primer (5 μmol/L) 0.8 μL, reverse primer (5 μmol/L) 0.8 μL, FastPfu DNA Polymerase 0.4 μL, template DNA 10 ng and DNase-free water. The PCR amplification was performed as follows: initial denaturation at 95 °C for 3 min, followed by 27 cycles of denaturing at 95 °C for 30 s, annealing at 60 °C for 30 s and extension at 72 °C for 45 s, and single extension at 72 °C for 10 min, and end at 4 °C (T100 Thermal Cycler PCR thermocycler, BIO-RAD, USA). After electrophoresis, the PCR products were purified using the AMPure^®^ PB beads (Pacifc Biosciences, CA, USA) and quantified with Qubit 4.0 (Thermo Fisher Scientific, USA). Purified products were pooled in equimolar and DNA library was constructed using the SMRTbell prep kit 3.0 (Pacifc Biosciences, CA, USA) according to PacBio's instructions. Purified SMRTbell libraries were sequenced on the Pacbio Sequel IIe System (Pacifc Biosciences, CA, USA) by Majorbio Bio-Pharm Technology Co., Ltd. (Shanghai, China). High-fidelity (HiFi) reads were obtained from the subreads, generated using circular consensus sequencing via SMRT Link v11.0. HiFi reads were barcode-identified and length-filtered. For bacterial 16S rRNA gene, sequences with a length < 1,000 or > 1,800 bp were removed. The optimized-HiFi reads were clustered into operational taxonomic units (OTUs) using UPARSE 7.1 with 97% sequence similarity level. The most abundant sequence for each OTU was selected as a representative sequence. The OTU table was manually filtered, i.e., chloroplast sequences in all samples were removed. To minimize the effects of sequencing depth on alpha and beta diversity measure, the number of 16S rRNA gene sequences from each sample were rarefied to 6,000 which still yielded an average Good’s coverage of 99.09%, respectively.

All data analyses were conducted on the Majorbio Cloud Platform (https://cloud.majorbio.com). Based on the OTUs information, rarefaction curves and alpha diversity indices including observed OTUs, Chao1 richness, Shannon index and Good’s coverage were calculated with Mothur v1.30.1. The similarity among the microbial communities in different samples was determined by principal coordinate analysis (PCoA) and non-metric multidimensional scaling (NMDS) based on Bray–curtis dissimilarity using Vegan v2.5–3 package. The PERMANOVA test was used to assess the percentage of variation explained by the treatment along with its statistical significance using Vegan v2.5–3 package. The linear discriminant analysis (LDA) effect size (LEfSe) (http://huttenhower.sph.harvard.edu/LEfSe) was performed to identify the significantly abundant taxa (phylum to genera) of bacteria among the different groups (LDA score > 2, *P* < 0.05). Based on the Spearman correlation coefficient |*r*| > 0.5 and *P* < 0.05, the species were selected for the analysis of the correlation network diagram.

Metabolome analysis was conducted using ultra-performance liquid chromatography tandem mass spectrometry (UPLC-Orbitrap Exploris 240, Thermo Fisher Scientific, Waltham, MA, USA). Approximately 100 μL of the fluid samples were transferred into centrifuge tubes (1.5 mL) and mixed with 300 μL of methanol and 10 μL of internal standard (2.8 mg/mL DL-o-Chlorophenylalanine). Then, the mixture was vortexed for 30 s (Vortex-5, Kylin-Bell Lab Instruments Co., Ltd., Haimen, China), kept for 1 h, and centrifuged at 13,000 × *g* and 4 °C for 15 min. In the end, 200 μL of supernatant was transferred into a vial for liquid chromatography tandem mass spectrometry analysis. The data were analyzed through the free online platform of Majorbio Cloud Platform (https://cloud.majorbio.com/page/tools). Orthogonal partial least squares discriminant analysis (OPLS-DA) with minimal supervision was conducted to reduce and classify the collected metabolomics data. The model’s validity was evaluated using model parameters R^2^X, R^2^Y, and Q^2^, which provide information on the interpretability and predictability, respectively, and help avoid the risk of over-fitting. Statistically significant differences among groups were identified with a VIP value greater than 1 and a *P* value less than 0.05. The differential metabolites were further identified and validated through KEGG. Enrichment analysis of the metabolic pathways was performed based on the differential metabolites using the KEGG pathway database (Database number: kegg_v20230830). KEGG pathway enrichment analysis was performed using relative-betweenness centrality method. The *P*-values were corrected for multiple testing at the pathway level using the FDR method (Benjamini-Hochberg). In the ruminal and jejunal metabolite analysis, the VIP value from multivariable OPLS-DA analysis and the *P* value from the univariable analysis *t*-test were used to screen for significantly differential metabolites (R software, Version 1.6.2). Metabolic pathway and enrichment analyses were then conducted on MetaboAnalyst 3.0 using the differential metabolites.

### Tissue sampling and PCR analysis

The subcutaneous fat, longissimus thoracis muscles and jejunum tissues were aseptically collected, immediately snap-frozen in liquid nitrogen, and subsequently stored at −80 °C for downstream RNA extraction and PCR analyses. Total RNA was extracted from tissue using the SteadyPure RNA Extraction Kit (AG21024, Accurate Biotechnology Co., Ltd., Hunan, China). RNA concentration and purity were assessed with a NanoDrop 2000 spectrophotometer (Thermo Fisher Scientific, USA), and integrity was verified via 1.0% agarose-formaldehyde gel electrophoresis. cDNA synthesis was performed using a reverse transcription kit (AG11707, Accurate Biotechnology Co., Ltd., Hunan, China). Primer sequences are listed in Table S1. Gene expression was quantified using a qRT-PCR kit (AG11701, Accurate Biotechnology Co., Ltd., Hunan, China) on a Bio-Rad CFX-96 system. Reactions were run in triplicate. Relative mRNA expression levels were normalized to the reference gene β-actin using the 2 ^− ΔΔCt^ method. Quantitative real-time PCR was performed in accordance with the Minimum Information for Publication of Quantitative Real-Time PCR Experiments guidelines. The reference gene β-actin was selected from a panel of candidate genes based on its superior expression stability across all experimental conditions, as determined by the geNorm algorithm.

### Statistical analysis

Data analysis was performed with a single beef cattle as the experimental unit. Data on animal growth performance, rumen fermentation, jejunal internal environment indicators, meat quality traits and blood biochemical and hormone indicators were analyzed using one-way ANOVA (SPSS 22.0 software, SPSS Inc., Chicago, IL, USA). A fixed-effects model was adopted for statistical analysis, in which treatment was specified as the only fixed factor. When the ANOVA indicated significant differences, post-hoc multiple comparisons were performed using Duncan’s test. Data visualization was performed by GraphPad Prism 8.0.2. Differences were considered significant when *P* < 0.05, and tendencies were considered when 0.05 < *P* < 0.10. The results are presented as mean values and standard error of the mean (SEM).

## Results

### Growth performance and apparent nutrient digestibility analysis

Table 2 illustrates the effects of supplementing with RUG and RPG on growth performance and apparent nutrient digestibility. RUG exhibited greater DMI compared with both CON and RPG groups (*P* = 0.001). Both the RUG and RPG groups demonstrated increased chest width (*P* = 0.001), chest girth (*P* = 0.013), and abdominal girth (*P* = 0.002) relative to CON. The RPG group showed superior apparent digestibility of EE (*P* = 0.027) and NDF (*P* = 0.027) compared to the other groups.

**Table 2:** Effects of dietary supplementation of rumen-protected and unprotected glucose on production performance and nutrient apparent digestibility of Xinjiang Brown cattle

Item	Treatments^1^			SEM	*P-*value
	CON	RUG	RPG		
Production performance indices					
Initial weight, kg	406.78	414.90	408.11	3.481	0.601
Final weight, kg	481.00	478.50	477.33	4.164	0.941
Average daily gain, kg	1.06	0.91	0.99	0.043	0.359
Dry matter intake, kg/d	10.14^b^	10.47^a^	10.07^b^	0.054	0.001
Feed conversion ratio	10.00	12.63	10.35	0.581	0.124
Body size indices, cm					
Withers heigh	128.89	128.40	131.33	0.685	0.179
Oblique body length	159.89	156.90	156.89	0.967	0.365
Chest depth	66.33	67.20	66.67	0.601	0.845
Chest width	40.11^b^	44.9^a^	45.44^a^	0.687	0.001
Chest girth	188.44^b^	196.6^a^	195.78^a^	1.298	0.013
Abdominal girth	213.67^c^	225.2^a^	219.67^b^	1.469	0.002
Cannon bone girth	20.33	21.30	20.89	0.203	0.149
Apparent nutrient digestibility, %					
Dry matter	80.89	81.59	82.92	0.371	0.080
Crude protein	61.95	62.65	61.91	0.517	0.805
Ether extract	57.23^b^	58.34^b^	64.19^a^	1.159	0.027
Neutral detergent fiber	60.91^b^	59.87^b^	63.83^a^	0.668	0.027
Acid detergent fiber	58.34	59.61	57.17	0.634	0.304

^a–c^Within a row, means with different superscript letters differ significantly (*P* < 0.05). Data are presented as least squares means with *n* = 10 per treatment

^1^ CON: basal diet + 150 g/head/d palmitate coating; RUG: basal diet + 150 g/head/d palmitate coating + 150 g/head/d rumen-unprotected glucose; RPG: basal diet + 300 g/head/d rumen-protected glucose

### Slaughter performance, meat quality and fatty acid profile analysis

Slaughter performance parameters including carcass weight, dressing percentage, net meat percentage, liver weight, and showed no treatment-related differences (Table 3; *P* > 0.05). However, both RUG and RPG displayed elevated backfat thickness (*P* = 0.041), and omental fat weight (*P* = 0.047) compared to CON. Notably, the mesenteric fat weight was significantly higher in the RUG group than in the CON and RPG groups (*P* = 0.05). The perirenal fat weight also showed an increasing trend (*P* = 0.095).

**Table 3:** Effects of dietary supplementation of rumen-protected and unprotected glucose on slaughter performance and meat quality of Xinjiang Brown cattle

Item	Treatments			SEM	*P*-value
	CON	RUG	RPG		
Slaughter performance indices					
Carcass weight, kg	252.16	246.42	253.27	2.437	0.474
Dressing percentage, %	53.09	51.93	52.52	0.706	0.810
Net meat percentage, %	45.49	43.30	44.47	0.697	0.448
Backfat thickness, cm	1.06^b^	1.38^a^	1.40^a^	0.063	0.041
Liver weight, kg	5.450	5.997	6.005	0.119	0.061
Perirenal fat weight, kg	12.85	13.87	12.61	0.261	0.095
Omental fat weight, kg	5.41^b^	6.71^a^	7.00^a^	0.286	0.047
Mesenteric fat weight, kg	5.84^b^	7.28^a^	5.87^b^	0.308	0.050
Meat quality indices					
Loin eye area, cm^2^	60.89	65.60	62.50	1.35	0.355
Marbling score^1^	1.00^b^	1.30^a^	1.00^b^	0.059	0.048
Moisture content, %	70.26	69.46	69.93	0.257	0.453
Protein content, DM basis, %	80.52	79.31	79.92	0.557	0.676
Intramuscular fat content, DM basis, %	12.18	15.58	12.51	0.681	0.064
Drip loss, %	4.44^a^	3.52^b^	3.86^b^	0.144	0.022
Cooking loss, %	34.38	32.06	33.63	0.970	0.619
Shear force, N	79.34^a^	70.96^b^	74.77^ab^	1.398	0.041

^a,b^Within a row, means with different superscript letters differ significantly (*P* < 0.05). Data are presented as least squares means with *n* = 10 per treatment

CON: basal diet + 150 g/head/d palmitate coating; RUG: basal diet + 150 g/head/d palmitate coating + 150 g/head/d rumen-unprotected glucose; RPG: basal diet + 300 g/head/d rumen-protected glucose

^1^Marbling was assessed using the Japanese Beef Marbling Standard (BMS), which grades meat on a scale from A1 (poor) to A5 (excellent). For statistical analysis, BMS grades (A1 to A5) were converted to numerical scores of 1 to 5, respectively

While meat pH, lightness (L*), redness (a*), yellowness (b*), loin eye area, moisture content, protein content, and cooking loss of LT remained unaffected (*P* > 0.05), the RUG group demonstrated improved marbling score (*P* = 0.048), IMF content (*P* = 0.064), reduced drip loss (*P* = 0.022) and shear force (*P* = 0.041) compared to CON (Table 3; Fig. S1). Fatty acid profiling revealed RUG group-specific elevations in methyl myristate (C14:0; *P* = 0.042) and methyl pentadecanoate (C15:0; *P* = 0.014), whereas the RPG group showed increased monounsaturated fatty acids (*P* = 0.045) and methyl myristoleate (C14:1; *P* = 0.03) (Table 4).

**Table 4:** Effects of dietary supplementation of rumen-protected and unprotected glucose on fatty acid profile of Xinjiang Brown cattle

Item	Treatments^1^			SEM	*P*-value
	CON	RUG	RPG		
Saturated fatty acids	45.76	46.34	44.71	0.291	0.063
C14:0	2.09^b^	2.44^a^	2.25^ab^	0.070	0.042
C15:0	0.26^b^	0.29^a^	0.25^b^	0.010	0.014
C16:0	26.68	27.08	26.10	0.200	0.135
C17:0	0.66	0.76	0.69	0.020	0.127
C18:0	16.07	15.77	15.42	0.200	0.453
Monounsaturated fatty acids	47.77	48.24	49.67	0.375	0.108
C14:1	0.44	0.58	0.57	0.030	0.077
C16:1	3.18	3.34	3.40	0.080	0.507
C18:1	1.88	1.84	1.81	0.050	0.887
C18:1n9c	42.29	42.30	43.67	0.390	0.203
C24:1	0.24	0.18	0.20	0.010	0.263
Polyunsaturated fatty acids	6.47	5.42	5.62	0.304	0.340
C18:2 n-6t	0.21	0.20	0.19	0.010	0.325
C18:2 n-6c	3.57	3.00	3.12	0.160	0.330
C18:3 n-3	0.23	0.21	0.18	0.020	0.461
C20:4 n-6	0.33	0.33	0.29	0.010	0.349
C20:3 n-6	0.43	0.36	0.40	0.020	0.467
C20:3 n-3	1.47	1.13	1.25	0.090	0.299
C22:2	0.23	0.21	0.20	0.020	0.827

^a,b ^Within a row, means with different superscript letters differ significantly (*P* < 0.05). Data are presented as least squares means with *n* = 10 per treatment

^1^ CON: basal diet + 150 g/head/d palmitate coating; RUG: basal diet + 150 g/head/d palmitate coating + 150 g/head/d rumen-unprotected glucose; RPG: basal diet + 300 g/head/d rumen-protected glucose

The expression of key lipogenic regulators involved in subcutaneous fat metabolism was significantly upregulated in the RPG group compared to the other groups (Table 5), including acetyl‑CoA carboxylase 1 (*ACC1*; *P* < 0.001), sterol regulatory element-binding protein 1c (*SREBP-1c*; *P* = 0.050), peroxisome proliferator-activated receptor γ (*PPARγ*; *P* = 0.003), fatty acid-binding protein 4 (*FABP4*; *P* = 0.006), Farnesoid X receptor (*FXR*; *P* = 0.042) and Takeda G protein-coupled receptor 5 *(TGR5*; *P* = 0.004). In contrast, adipose triglyceride lipase (*ATGL*) expression was suppressed in the RUG group relative to the CON group (*P* = 0.048). Furthermore, compared with the CON group, the RUG group showed significant upregulation in the relative mRNA expression of the lipogenic genes *ACC1* (*P* = 0.035) and *FABP4* (*P* = 0.001) in the longissimus thoracis muscle, while *PPARγ* expression also exhibited an increasing trend (*P* = 0.090). Conversely, the RPG group displayed significant downregulation in the relative expression of *SREBP-1c* (*P* = 0.05) and *ATGL* (*P* = 0.002).

**Table 5:** Effects of dietary supplementation of rumen-protected and unprotected glucose on relative mRNA expression in subcutaneous fat and longissimus thoracis muscle of Xinjiang Brown cattle

Item	Treatments^1^			SEM	*P-*value
	CON	RUG	RPG		
Subcutaneous fat tissue					
*ACC1*	1.00^b^	1.00^b^	1.82^a^	0.105	< 0.001
*PPARγ*	1.00^b^	1.15^b^	2.40^a^	0.193	0.003
*SREBP-1C*	1.00^b^	0.94^b^	1.62^a^	0.129	0.050
*ATGL*	1.00^a^	0.61^b^	0.89^ab^	0.220	0.048
*CPT1*	1.00	0.65	0.79	0.148	0.641
*FABP4*	1.00^b^	1.30^ab^	1.52^a^	0.070	0.006
*FXR*	1.00^ab^	0.82^b^	1.26^a^	0.074	0.042
*TGR5*	1.00^b^	0.90^b^	1.75^a^	0.120	0.004
Longissimus thoracis muscle tissue					
*ACC1*	1.00^b^	3.20^a^	2.40^ab^	0.358	0.035
*PPARγ*	1.00	1.38	1.22	0.075	0.090
*SREBP-1C*	1.00^a^	0.75^b^	0.64^b^	0.042	0.001
*ATGL*	1.00^a^	0.81^ab^	0.58^b^	0.051	0.002
*CPT1*	1.00	0.54	0.94	0.106	0.162
*FABP4*	1.00^b^	2.07^a^	1.23^b^	0.118	0.001
*FXR*	1.00	0.89	0.96	0.154	0.962
*TGR5*	1.00	0.91	0.97	0.077	0.886

^a,b ^Within a row, means with different superscript letters differ significantly (*P* < 0.05). Data are presented as least squares means with *n* = 10 per treatment

^1^ CON: basal diet + 150 g/head/d palmitate coating; RUG: basal diet + 150 g/head/d palmitate coating + 150 g/head/d rumen-unprotected glucose; RPG: basal diet + 300 g/head/d rumen-protected glucose

*ACC1* Acetyl-coA carboxylase 1, *PPARγ* Peroxisome proliferator-activated receptor γ, *SREBP-1C* Sterol regulatory element-binding protein 1C, *ATGL* Adipose triglyceride lipase, *CPT1* Carnitine palmitoyltransferase 1, *FABP4* Fatty acid binding protein 4, *FXR* Farnesoid X receptor, *TGR5* G protein-coupled bile acid receptor 1

### Serum biochemistry and hormone indicators analysis

Serum biochemistry parameters revealed higher circulating GLU (*P* = 0.037) and TG (*P* = 0.017) levels in RPG group compared to other groups (Table 6). Serum hormone analysis demonstrated that both RUG and RPG groups had increased INS (*P* < 0.001), GLP-1 (*P* < 0.001), cortisol (*P* = 0.001), LEP (*P* < 0.001), T4 (*P* = 0.004), EPI (*P* < 0.001) and 5-HT (*P* < 0.001) accompanied by decreased ADP (*P* < 0.001) levels compared with CON (Table 6). Triiodothyronine was uniquely elevated in RUG group than other groups (*P* < 0.001).

**Table 6:** Effects of dietary supplementation of rumen-protected and unprotected glucose on serum biochemical and hormonal indexes of Xinjiang Brown cattle

Item	Treatments^1^			SEM	*P-*value
	CON	RUG	RPG		
Serum biochemical indices, mmol/L					
Glucose	4.80^b^	4.75^b^	5.32^a^	0.103	0.037
Cholesterol	4.71	4.30	5.44	0.215	0.076
Triglycerides	0.20^b^	0.33^ab^	0.42^a^	0.032	0.017
Serum hormone indices					
Insulin, mIU/L	49.90^c^	57.26^a^	51.83^b^	0.608	< 0.001
Glucagon, ng/L	106.62	112.96	112.03	1.352	0.116
Glucagon-like peptide-1, ng/L	34.69^b^	41.82^a^	41.58^a^	0.914	< 0.001
Cortisol, μg/L	173.90^b^	210.66^a^	203.30^a^	5.016	0.001
Adiponectin, μg/L	209.98^a^	159.29^c^	172.72^b^	5.700	< 0.001
Leptin, μg/L	3.595^b^	4.277^a^	4.307^a^	0.093	< 0.001
Epinephrine, ng/L	91.45^c^	115.24^a^	107.79^b^	2.570	< 0.001
Thyroxine, pmol/L	1,063.69^b^	1,229.22^a^	1,184.37^a^	24.052	0.004
Triiodothyronine, pmol/L	84.14^b^	97.16^a^	86.14^b^	1.669	< 0.001
5-Hydroxytryptamine, ng/L	1,231.57^c^	3,009.14^a^	2,682.58^b^	133.528	< 0.001

^a–c^Within a row, means with different superscript letters differ significantly (*P* < 0.05). Data are presented as least squares means with *n* = 6 per treatment

^1^ CON: basal diet + 150 g/head/d palmitate coating; RUG: basal diet + 150 g/head/d palmitate coating + 150 g/head/d rumen-unprotected glucose; RPG: basal diet + 300 g/head/d rumen-protected glucose

### Rumen fermentation parameters, microbiota community and metabolites analysis

As shown in Table 7, no differences were detected among groups in ruminal pH, NH_3_-N, or MCP content (*P* > 0.05). The RUG group increased total volatile fatty acids (*P* = 0.045), acetate (*P* = 0.068) and propionate (*P* = 0.011) molar concentrations compared to CON.

**Table 7:** Effects of dietary supplementation of rumen-protected and unprotected glucose on rumen fermentation of Xinjiang Brown cattle

Item	Treatments^1^			SEM	*P-*value
	CON	RUG	RPG		
pH ruminal	5.88	5.85	5.91	0.027	0.673
NH_3_-N, mg/100 mL	8.15	7.50	7.67	0.037	0.790
MCP, mg/100 mL	110.76	104.17	103.87	2.669	0.517
Total VFA, mmol/L	184.77^b^	202.33^a^	189.21^ab^	3.102	0.045
VFA profile, mmol/L					
Acetate	142.64	153.65	147.47	1.992	0.068
Propionate	25.30^b^	28.99^a^	26.54^b^	0.543	0.011
Butyrate	14.09	15.96	16.27	0.456	0.112
Valerate	1.29	1.38	1.35	0.070	0.888
Isobutyrate	1.09	1.12	0.91	0.056	0.255
Isovalerate	1.32	1.24	1.26	0.069	0.897
Acetate to propionate ratio	5.57	5.38	5.63	0.099	0.585

^a,b ^Within a row, means with different superscript letters differ significantly (*P* < 0.05). Data are presented as least squares means with *n* = 10 per treatment

^1^ CON: basal diet + 150 g/head/d palmitate coating; RUG: basal diet + 150 g/head/d palmitate coating + 150 g/head/d rumen-unprotected glucose; RPG: basal diet + 300 g/head/d rumen-protected glucose

*MCP* Microbial crude protein, *VFA* Volatile fatty acids

**Fig. 1:**
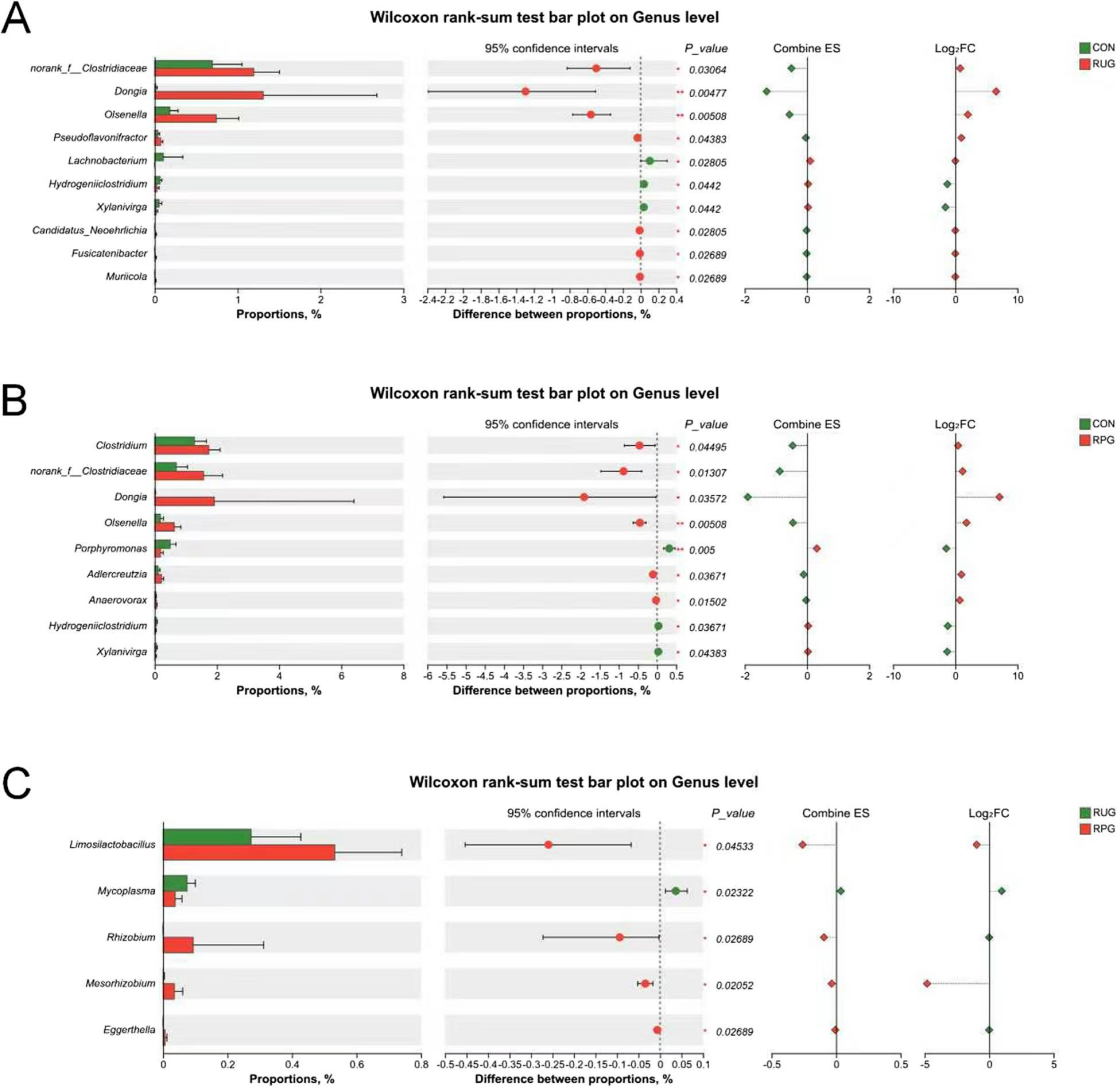
The differences in relative abundance at the genus level in rumen fluid samples of Xinjiang Brown cattle among treatment groups. **A** Differential microorganisms between CON and RUG groups (genus level). **B** Differential microorganisms between CON and RPG groups (genus level). **C** Differential microorganisms between RUG and RPG groups (genus level). Significance levels are denoted as ^*^P < 0.05, ^**^P < 0.01 and ^***^P < 0.001. CON: basal diet + 150 g/head/d palmitate coating; RUG: basal diet + 150 g/head/d palmitate coating + 150 g/head/d rumen-unprotected glucose; RPG: basal diet + 300 g/head/d rumen-protected glucose

**Fig. 2:**
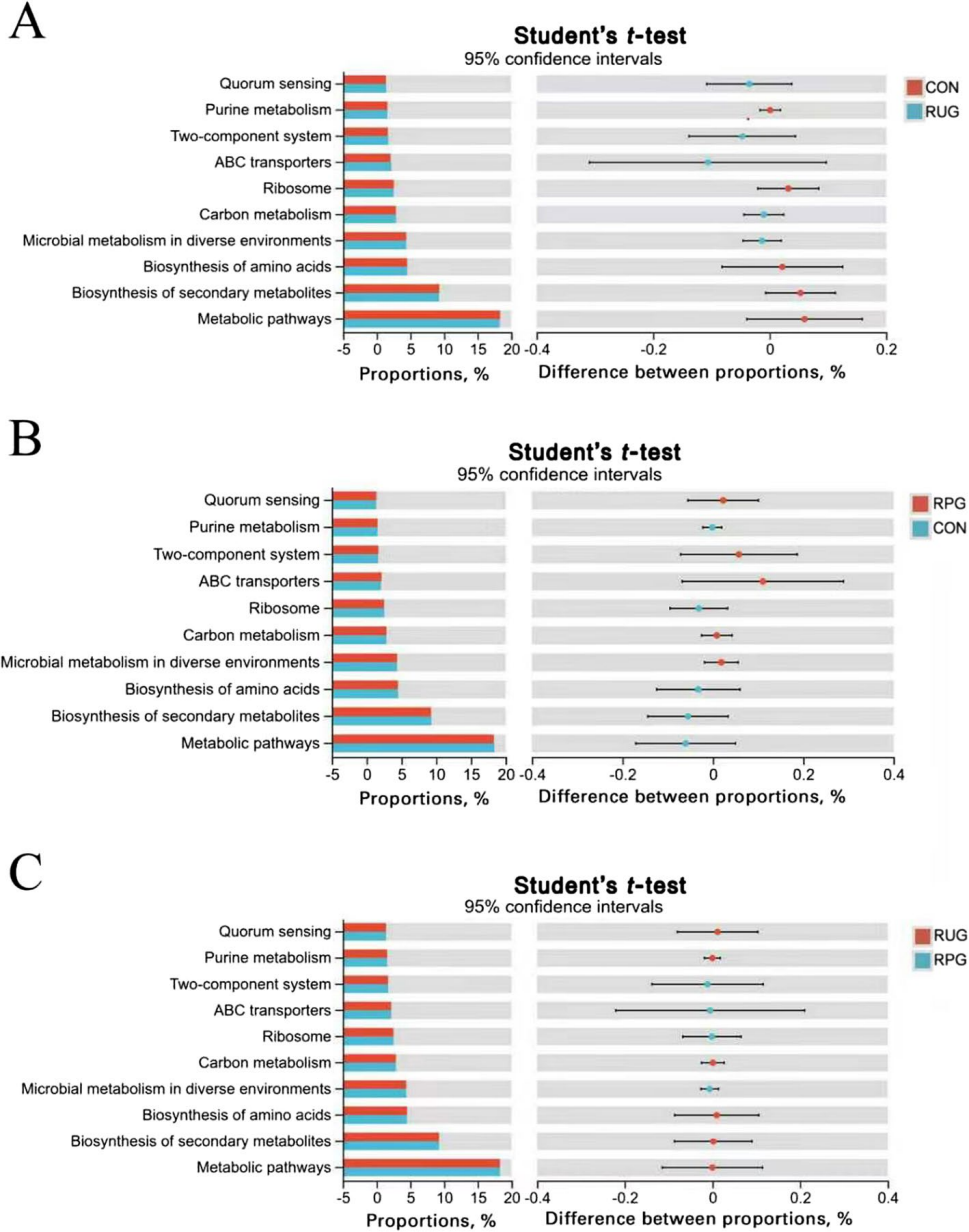
Functional prediction of rumen differential microorganisms between treatment groups. Significance levels are denoted as ^*^P < 0.05, ^**^P < 0.01 and ^***^P < 0.001. CON: basal diet + 150 g/head/d palmitate coating; RUG: basal diet + 150 g/head/d palmitate coating + 150 g/head/d rumen-unprotected glucose; RPG: basal diet + 300 g/head/d rumen-protected glucose

**Fig. 3:**
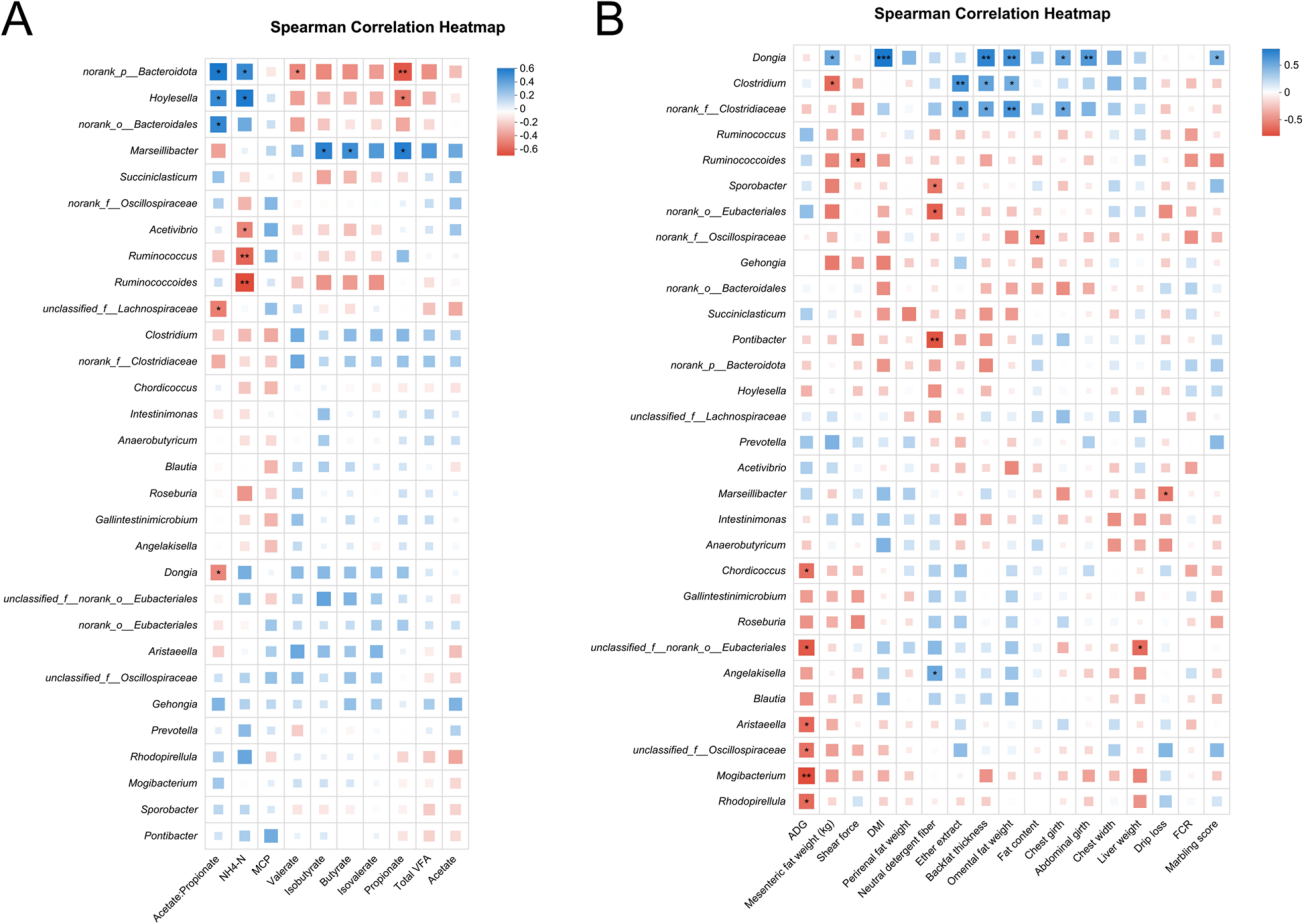
Correlation analysis of rumen bacteria with rumen fermentation parameters and apparent difference index. **A** Spearman’s rank correlation analysis between the top 30 bacteria at the genus level and rumen fermentation parameters. **B** Spearman’s rank correlation analysis between the top 30 bacteria at the genus level and apparent difference index. Significance levels are denoted as ^*^P < 0.05, ^**^P < 0.01 and ^***^P < 0.001

Rarefaction curves demonstrated that species richness (Sobs) stabilized at 13,000 sequences, with Good’s Coverage values exceeding 0.99 (Fig. S2A and B), confirming sufficient sequencing depth and data accuracy. Observed OTU counts were 1,332, 1,270, and 1,283 for CON, RUG, and RPG groups, respectively (Fig. S3). Alpha diversity indices (Shannon, Chao, ACE, Simpson) showed no significant effects among treatment groups (*P* > 0.05; Fig. S4). Beta diversity analyses (PCoA, NMDS) revealed no structural divergence in microbial communities across groups (*P* > 0.05; Fig. S5). Taxonomic profiling revealed Bacillota (75.02%), Bacteroidota (18.51%), and Pseudomonadota (2.54%) as dominant phyla (Fig. S6A), with *Aristaeella* (13.69%), *Ruminococcus* (6.85%), and *Gallintestinimicrobium* (6.04%) constituting core genera (Fig. S6B). Differential abundance analysis using Wilcoxon rank-sum tests revealed distinct microbial shifts across treatment groups (Fig. 1). Compared to CON, RUG group exhibited significantly increased relative abundances of *norank_f__Clostridiaceae*, *Dongia*, *Olsenella*, and *Pseudoflavonifractor*, but decreased *Lachnobacterium*, *Hydrogeniiclostridium*, and *Xylanivirga* (*P* < 0.05; Fig. 1A). Compared to CON, RPG group showed elevated *Clostridium, norank_f__Clostridiaceae*, *Dongia*, and *Olsenella*, with reduced *Porphyromonas* (*P* < 0.05; Fig. 1B). Compared to RUG group, RPG group displayed higher *Limosilactobacillus*, *undefined_Chloroplast*, *Rhizobium*, and *Mesorhizobium*, but lower *Mycoplasma* (*P* < 0.05; Fig. 1C). LEfSe analysis identified group-specific biomarkers with LDA scores > 3: *Porphyromonas*, *Eggerthella*, *Lachnobacterium*, and *Xylanivirga* for CON; *Olsenella* and *Mycoplasma* for RUG group; and *norank_f__Clostridiaceae*, *Dongia*, and *Rhizobium* for RPG group (Fig. S7). Functional prediction analysis based on the KEGG database revealed that the functions of the rumen microbial community were primarily enriched in metabolic pathways such as secondary metabolite biosynthesis, amino acid biosynthesis, and carbon metabolism. Welch’s *t*-test results indicated that no significant effects were observed in the enrichment levels of these pathways, including secondary metabolite biosynthesis, amino acid biosynthesis, and carbon metabolism, among the groups (*P* > 0.05; Fig. 2). Correlation analysis of the top 30 bacterial genera with ruminal fermentation parameters and performance indices revealed that *norank_p__Bacteroidota* and *Hoylesella* were negatively correlated with ruminal propionate molar concentration while *Marseillibacter* showed a positive correlation (Fig. 3A; *P* < 0.05). *Norank_p__Bacteroidota*, *Hoylesella*, and *norank_o__Bacteroidales* correlated positively with the acetate-to-propionate ratio, whereas *Dongia* correlated negatively (Fig. 3A; *P* < 0.05). *Dongia* exhibited positive correlations with DMI, chest girth, abdominal girth, marbling score, and mesenteric fat weight, while *Clostridium* was negatively correlated with mesenteric fat weight (Fig. 3B; *P* < 0.05). *Dongia*, *Clostridium*, and *norank_f__Clostridiaceae* showed positive correlations with backfat thickness and omental fat weight (Fig. 3B; *P* < 0.05).

**Fig. 4:**
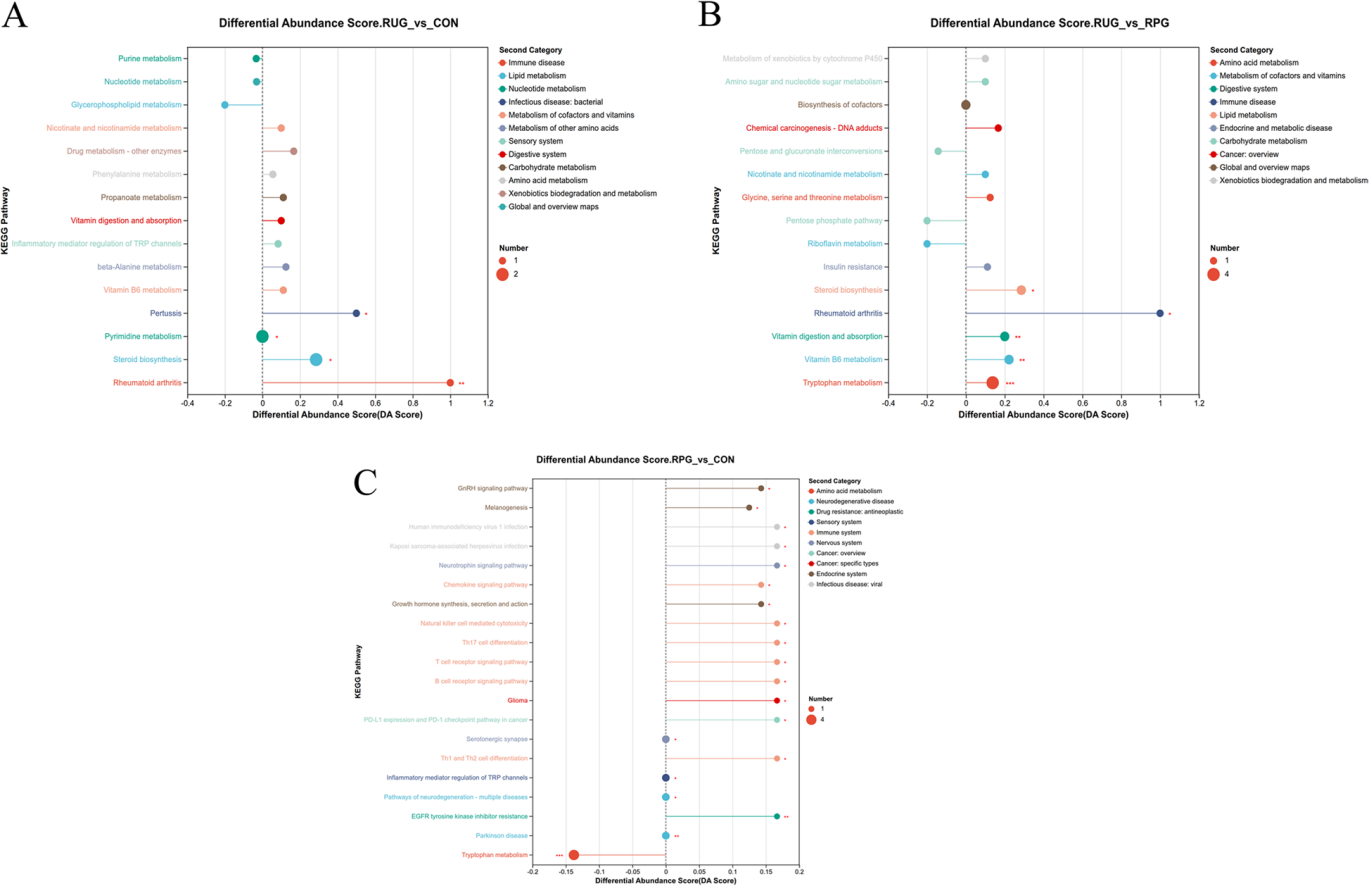
The differential pathways enriched by differential metabolic ensembles in rumen fluid samples of Xinjiang Brown cattle. **A** KEGG metabolic pathway enriched by RUG vs. CON metabolic set. **B** KEGG metabolic pathway enriched by RUG vs. RPG metabolic set. **C** KEGG metabolic pathway enriched by RPG vs. CON metabolic set. Significance levels are denoted as ^*^P < 0.05, ^**^P < 0.01 and ^***^P < 0.001. CON: basal diet + 150 g/head/d palmitate coating; RUG: basal diet + 150 g/head/d palmitate coating + 150 g/head/d rumen-unprotected glucose; RPG: basal diet + 300 g/head/d rumen-protected glucose

**Fig. 5:**
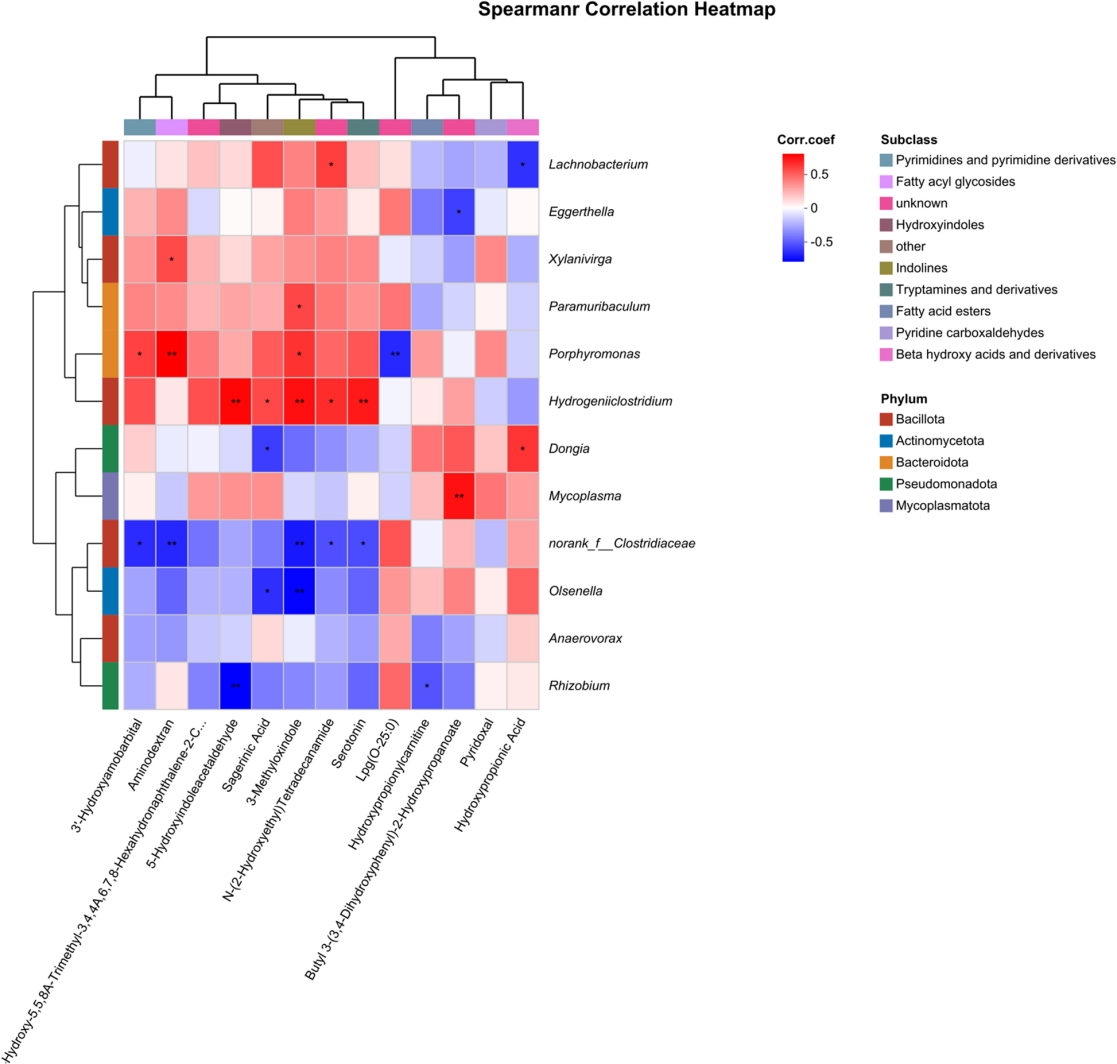
Correlation analysis of key microorganisms and metabolites in rumen fluid of Xinjiang Brown cattle. Significance levels are denoted as ^*^P < 0.05, ^**^P < 0.01 and ^***^P < 0.001

We employed the Student’s *t*-test method to identify 115, 167, and 271 differentially abundant metabolites in the RUG group vs. CON, the RUG group vs. RPG group and the RPG group vs. CON, respectively (VIP > 1, fold change > 1, *P* < 0.05; Fig. S8). The KEGG enrichment analysis demonstrated distinct pathway activation patterns across treatment comparisons (Fig. 4). The RUG group vs. CON metabolic set showed enrichment in propanoate metabolism, glycerophospholipid metabolism, phenylalanine metabolism, and vitamin digestion and absorption, with significant enrichment in steroid biosynthesis (Fig. 4A). The RUG group vs. RPG group metabolic set revealed predominant involvement of pentose and glucuronate interconversions, riboflavin metabolism, while enrichment in steroid biosynthesis and tryptophan metabolism (Fig. 4B). Notably, the RPG group vs. CON metabolic set exhibited enrichment in growth hormone regulation pathways, MAPK signaling, NF-κB signaling and tryptophan metabolism (Fig. 4C). Kruskal–Wallis H tests identified 13 key rumen metabolites (Table 8). For the between-group comparison of metabolomics data, we first calculated the raw *P*-values at the metabolite level. Subsequently, to control the false positive risk arising from multiple comparisons, we performed Benjamini–Hochberg false discovery rate (FDR) correction to obtain adjusted *q*-values. Given that all adjusted *q*-values were greater than 0.05, the metabolomics results in this section should be regarded as exploratory analysis, aiming to identify potential changing trends and to generate hypotheses for follow-up studies. Compared to CON, RUG group exhibited elevated hydroxypropionic acid abundance (*P* = 0.042), while reduced relative abundances of sagerinic acid (*P* = 0.045), 3-methyloxindole (*P* = 0.025), and N-(2-hydroxyethyl)-tetradecanamide (*P* = 0.019). RPG group displayed decreased relative abundance of aminodextran (*P* = 0.049), 3'-hydroxyamobarbital (*P* = 0.030), serotonin (*P* = 0.032), and 5-hydroxyindoleacetaldehyde (*P* = 0.030) than CON. The relative abundance of hydroxypropionylcarnitine (*P* = 0.042), butyl 3-(3,4-dihydroxyphenyl)-2-hydroxypropanoate (*P* = 0.029), and pyridoxal (*P* = 0.038) were higher in RUG group than RPG group, whereas LPG (O-25:0; *P* = 0.035) and 3-hydroxy-5,5,8a-trimethyl-3,4,4a,6,7,8-hexahydronaphthalene-2-carboxylic acid (*P* = 0.023) was lower. These metabolites were enriched in tryptophan metabolism and propanoate metabolism pathways (Fig. S9). Integrated Spearman correlation network (Fig. 5) unveiled critical microbiome-metabolome linkages. *Dongia* was positively correlated with hydroxypropionic acid but was negatively correlated with sagerinic acid (*P* < 0.05). The *norank_f__Clostridiaceae* was negatively correlated with 3'-hydroxyamobarbital, aminodextran, 3-methyloxindole, N-(2-hydroxyethyl), tetradecanamide, and serotonin (*P* < 0.05). *Olsenella* was negatively correlated with sagerinic acid and 3-methyloxindole (*P* < 0.05). *Mycoplasma* was positively correlated with butyl 3-(3,4-dihydroxyphenyl)-2-hydroxypropanoate, while *Rhizobium* was negatively correlated with 5-hydroxyindoleacetaldehyde and hydroxypropionylcarnitine (*P* < 0.05).

**Table 8:** Relative abundance of key differential metabolites in rumen between treatment groups

Item	Treatment			SEM	*P*-value	*q*-value
	CON	RUG	RPG			
Sagerinic acid	4.41^a^	3.87^b^	3.78^b^	0.189	0.045	> 0.100
Aminodextran	5.08^a^	4.90^a^	4.58^b^	0.114	0.049	> 0.100
Hydroxypropionylcarnitine	4.94^ab^	5.14^a^	4.87^b^	0.073	0.042	> 0.100
3-Methyloxindole	5.78^a^	5.60^b^	5.58^b^	0.041	0.025	> 0.100
Lpg(O-25:0)	5.64^ab^	5.62^b^	5.77^a^	0.036	0.035	> 0.100
Butyl 3-(3,4-dihydroxyphenyl)-2-hydroxypropanoate	4.30^ab^	4.38^a^	4.25^b^	0.030	0.029	> 0.100
N-(2-Hydroxyethyl)Tetradecanamide	5.79^a^	5.69^b^	5.67^b^	0.025	0.019	> 0.100
3'-Hydroxyamobarbital	5.29^a^	5.30^a^	5.18^b^	0.029	0.030	> 0.100
Hydroxypropionic acid	5.16^b^	5.26^a^	5.21^ab^	0.023	0.029	> 0.100
3-Hydroxy-5,5,8A-trimethyl-3,4,4A,6,7,8-hexahydronaphthalene-2-carboxylic acid	4.70^b^	4.62^b^	4.70^a^	0.019	0.023	> 0.100
Serotonin	5.01^a^	4.96^a^	4.89^b^	0.029	0.032	> 0.100
5-Hydroxyindoleacetaldehyde	4.50^a^	4.49^a^	4.41^b^	0.021	0.030	> 0.100
Pyridoxal	5.27^ab^	5.32^a^	5.23^b^	0.018	0.038	> 0.100

^a,b^Within a row, means with different superscript letters differ significantly (*P* < 0.05). Data are presented as least squares means with *n* = 6 per treatment

CON: basal diet + 150 g/head/d palmitate coating; RUG: basal diet + 150 g/head/d palmitate coating + 150 g/head/d rumen-unprotected glucose; RPG: basal diet + 300 g/head/d rumen-protected glucose

### Jejunal environmental parameters, microbiota community and metabolites analysis

As shown in Table 9, RUG group exhibited higher acetate and propionate molar concentrations compared to other groups, while RPG group displayed higher acetate than CON (*P* < 0.05). LCA content in RUG group was significantly reduced relative to other groups, whereas RPG group showed significantly higher LCA than CON (Table 9; *P* < 0.05). Both RUG and RPG groups exhibited significantly decreased DCA levels compared to CON (Table 9; *P* < 0.05).

**Table 9:** Effects of dietary supplementation of rumen-protected and unprotected glucose on jejunum VFA, secondary bile acid content and relative mRNA expression in jejunal epithelial of Xinjiang Brown cattle

Item	Treatments^1^			SEM	*P-*value
	CON	RUG	RPG		
pH jejunal	6.78	6.73	6.55	0.075	0.440
Acetate, mmol/L	13.75^c^	21.64^a^	16.78^b^	0.839	< 0.001
Propionate, mmol/L	0.48^b^	0.69^a^	0.45^b^	0.032	0.001
Butyrate, mmol/L	0.76	0.68	0.60	0.059	0.562
Lithocholic acid, ng/L	71.57^b^	48.55^c^	77.81^a^	2.241	< 0.001
Deoxycholic acid, ng/L	131.88^a^	88.65^b^	79.42^c^	4.235	< 0.001
*FXR*	1.00^b^	1.97^a^	0.84^b^	0.147	0.001
*TGR5*	1.00	1.01	1.59	0.146	0.166

^a–c^Within a row, means with different superscript letters differ significantly (*P* < 0.05). Data are presented as least squares means with *n* = 10 per treatment

^1^ CON: basal diet + 150 g/head/d palmitate coating; RUG: basal diet + 150 g/head/d palmitate coating + 150 g/head/d rumen-unprotected glucose; RPG: basal diet + 300 g/head/d rumen-protected glucose

*FXR* Farnesoid X receptor, *TGR5* G protein-coupled bile acid receptor 1

**Fig. 6:**
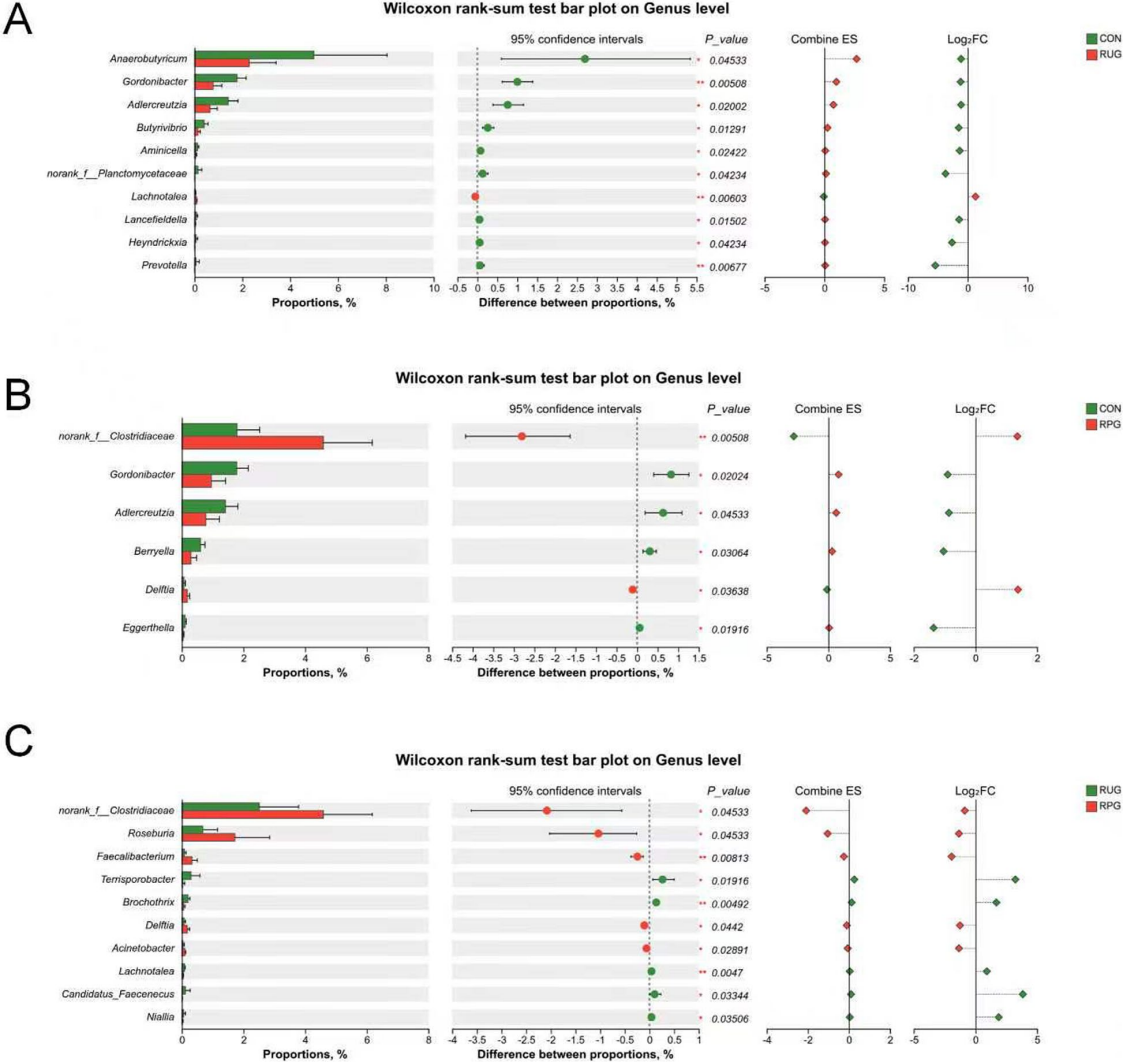
The differences in relative abundance at the genus level in jejunal fluid samples of Xinjiang Brown cattle among treatment groups. **A** Differential microorganisms between CON and RUG groups (genus level). **B** Differential microorganisms between CON and RPG groups (genus level). **C** Differential microorganisms between RUG and RPG groups (genus level). Significance levels are denoted as ^*^P < 0.05, ^**^P < 0.01 and ^***^P < 0.001. CON: basal diet + 150 g/head/d palmitate coating; RUG: basal diet + 150 g/head/d palmitate coating + 150 g/head/d rumen-unprotected glucose; RPG: basal diet + 300 g/head/d rumen-protected glucose

**Fig. 7:**
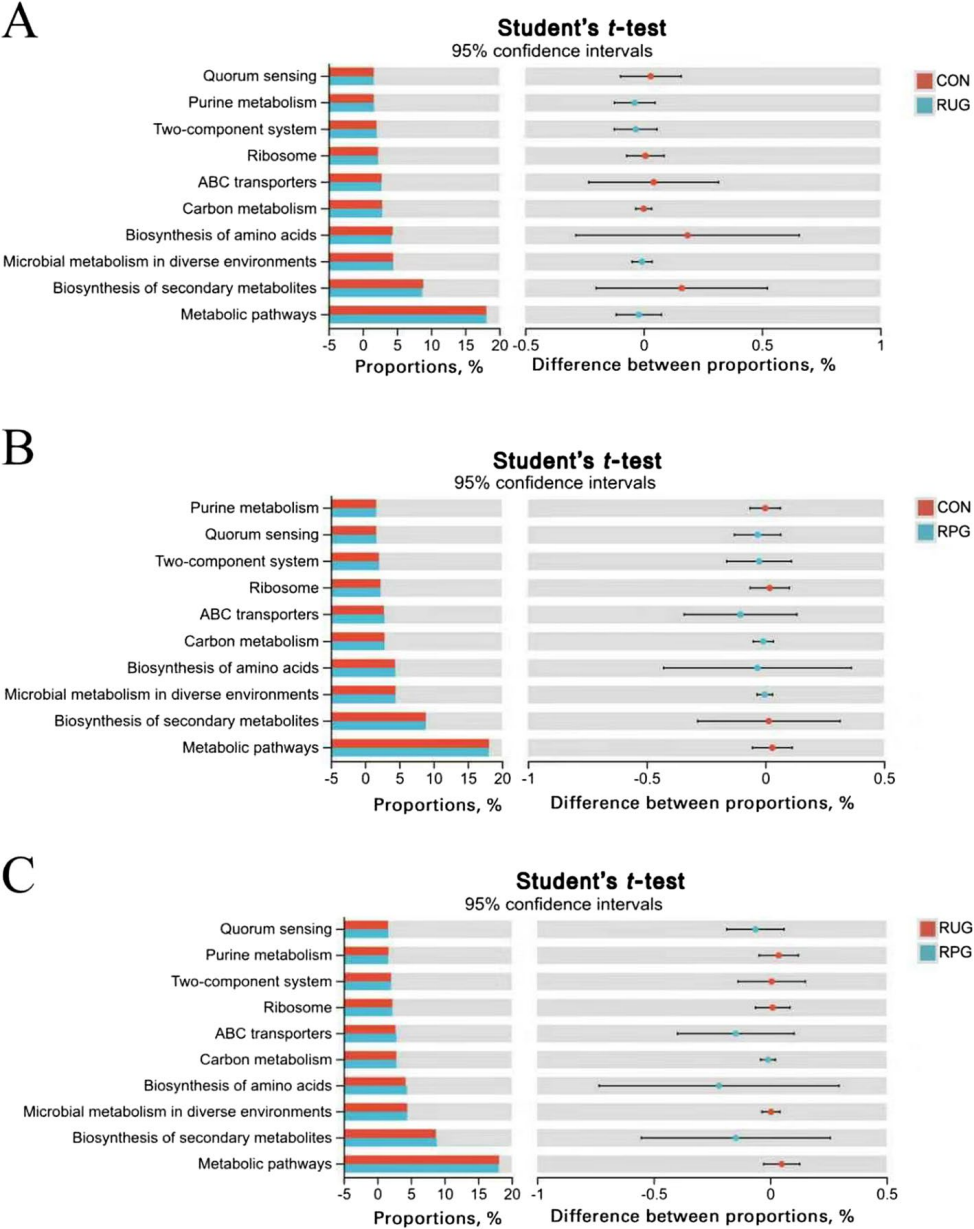
Functional prediction of differential microorganisms in jejunum between treatment groups. Significance levels are denoted as ^*^P < 0.05, ^**^P < 0.01 and ^***^P < 0.001. CON: basal diet + 150 g/head/d palmitate coating; RUG: basal diet + 150 g/head/d palmitate coating + 150 g/head/d rumen-unprotected glucose; RPG: basal diet + 300 g/head/d rumen-protected glucose

**Fig. 8:**
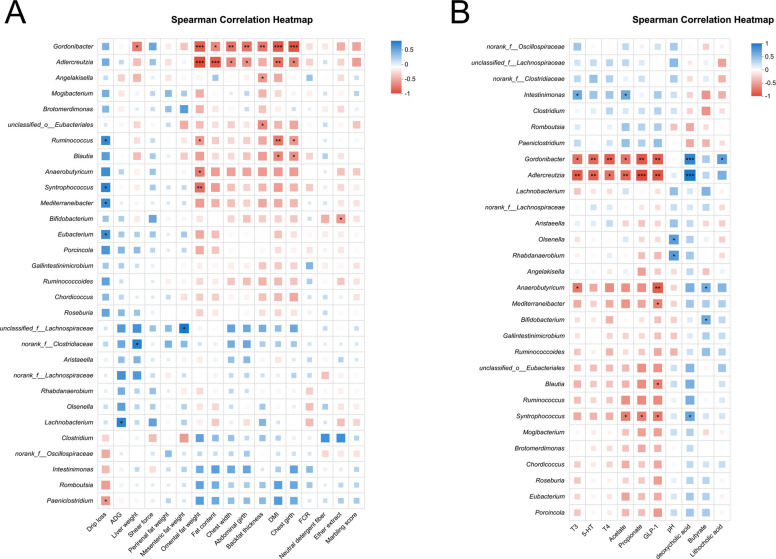
Correlation analysis of jejunal bacteria with jejunal fermentation parameters and apparent difference index. **A** Spearman’s rank correlation analysis between the top 30 bacteria at the genus level and apparent difference index. **B** Spearman’s rank correlation analysis between the top 30 bacteria at the genus level and jejunal fermentation parameters. Significance levels are denoted as ^*^P < 0.05, ^**^P < 0.01 and ^***^P < 0.001

Rarefaction curves indicated stable species richness (Sobs) at 11,000 sequences, with Good’s Coverage > 0.99 (Fig. S10), confirming adequate sequencing depth and accuracy. Observed OTU counts were 633, 606, and 587 for CON, RUG, and RPG groups, respectively (Fig. S11). Alpha diversity indices (Shannon, Chao, ACE, Simpson) showed no significant effects among groups (Fig. S12; *P* > 0.05). Beta diversity analyses (PCoA, NMDS) revealed no structural divergence in microbial communities (Fig. S13; *P* > 0.05). Taxonomic profiling revealed Bacillota (89.37%), Actinomycetota (8.36%), and Pseudomonadota (0.84%) as dominant phyla (Fig. S14A), with *Gallintestinimicrobium* (3.34%), *Aristaeella* (3.06%), *unclassified_f__Lachnospiraceae* (2.84%), and *Romboutsia* (2.82%) constituting core genera (Fig. S14B). Differential abundance analysis revealed distinct microbial shifts across treatment groups. RUG group showed reduced relative abundances of *Anaerobutyricum*, *Gordonibacter*, *Adlercreutzia*, *Butyrivibrio*, and *Prevotella*, but increased *Lachnotalea* compared to CON (Fig. 6A; *P* < 0.05). RPG group exhibited higher relative abundances of *norank_f__Clostridiaceae* and lower *Gordonibacter*, *Adlercreutzia*, and *Berryella* versus CON (Fig. 6B; *P* < 0.05). Compared to RUG group, RPG group displayed elevated relative abundances of *norank_f__Clostridiaceae*, *Roseburia*, and *Faecalibacterium* but reduced *Lachnotalea*, *Terrisporobacter*, and *Brochothrix* (Fig. 6C; *P* < 0.05). LEfSe analysis highlighted *Anaerobutyricum*, *Gordonibacter*, and *Adlercreutzia* as CON biomarkers; *Lachnotalea* for RUG group; and *norank_f__Clostridiaceae* for RPG group (Fig. S15). Functional prediction analysis based on the KEGG database revealed that the functions of the jejunal microbial community were primarily enriched in metabolic pathways such as secondary metabolite biosynthesis, amino acid biosynthesis, and carbon metabolism. Welch’s *t*-test results indicated that no significant effects were observed in the enrichment levels of these pathways-including secondary metabolite biosynthesis, amino acid biosynthesis, and carbon metabolism-among the groups (*P* > 0.05; Fig. 7). Correlation analyses revealed *Gordonibacter* and *Adlercreutzia* were negatively associated with DMI, chest width, chest girth, abdominal girth, omental fat weight, backfat thickness, and IMF content (Fig. 8A; *P* < 0.05). The *unclassified_f__Lachnospiraceae* was correlated with mesenteric fat weight (Fig. 8A; *P* < 0.05). *Gordonibacter* and *Adlercreutzia* were negatively correlated with jejunal acetate, propionate, blood 5-HT, GLP-1, T3, and T4, but were positively correlated with DCA and LCA (Fig. 8B; *P* < 0.05).

**Fig. 9:**
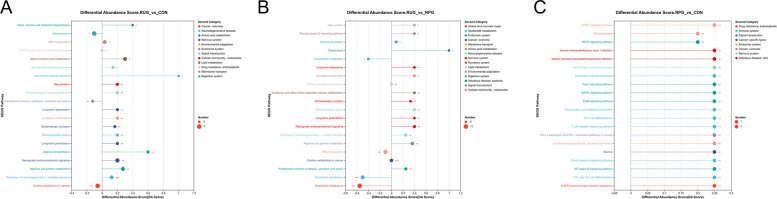
The differential pathways enriched by differential metabolic ensembles in jejunal fluid samples of Xinjiang Brown cattle. **A** KEGG metabolic pathway enriched by RUG vs. CON metabolic set. **B** KEGG metabolic pathway enriched by RUG vs. RPG metabolic set. **C** KEGG metabolic pathway enriched by RPG vs. CON metabolic set. Significance levels are denoted as ^*^P < 0.05, ^**^P < 0.01 and ^***^P < 0.001. CON: basal diet + 150 g/head/d palmitate coating; RUG: basal diet + 150 g/head/d palmitate coating + 150 g/head/d rumen-unprotected glucose; RPG: basal diet + 300 g/head/d rumen-protected glucose

**Fig. 10:**
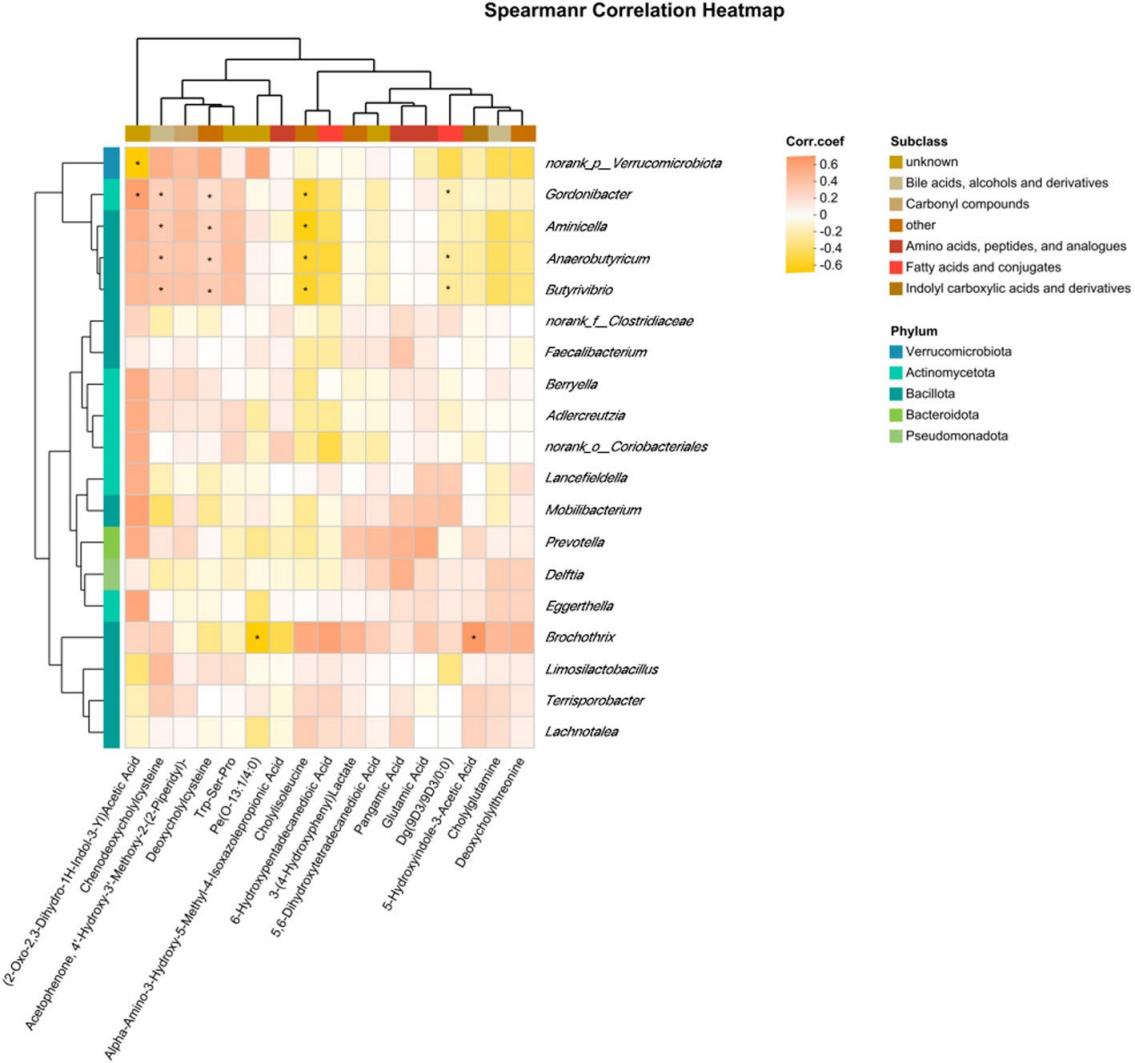
Correlation analysis of key microorganisms and metabolites in jejunal fluid of Xinjiang Brown cattle. Significance levels are denoted as ^*^P < 0.05, ^**^P < 0.01 and ^***^P < 0.001

We employed the Student’s *t*-test method to identify 542, 624, and 72 differentially abundant metabolites in the RUG group vs. CON, the RUG group vs. RPG group and the RPG group vs. CON, respectively (Fig. S16). KEGG enrichment analysis demonstrated distinct pathway activation patterns across treatment comparisons (Fig. 9). The RUG group vs. CON metabolic set showed enrichment in bile secretion, alpha-linolenic acid metabolism, parathyroid hormone synthesis, and valine/leucine/isoleucine biosynthesis (Fig. 9A). The RUG group vs. RPG group metabolic set revealed enrichment in cholesterol metabolism, mineral absorption, primary bile acid biosynthesis, and glutamatergic synapse (Fig. 9B). Notably, the RPG group vs. CON metabolic set exhibited enrichment in GnRH signaling, melanogenesis, growth hormone synthesis, MAPK signaling, and NF-κB signaling (Fig. 9C). Kruskal–Wallis tests identified 17 key metabolites (Table 10). Among the key metabolites in the jejunum, the *q*-values of Dg(9D3/9D3/0:0), deoxycholylcysteine, deoxycholylthreonine, and cholylisoleucine were less than 0.05 between groups, while the *q*-values of the other key metabolites were all greater than 0.05. Metabolites with *q*-values greater than 0.05 are considered exploratory, aiming to identify potential changing trends and to generate hypotheses for subsequent research. Both rumen-protected and RUG groups showed significantly elevated relative abundances of cholylisoleucine (*P* = 0.003), DG(9D3/9D3/0:0) (*P* = 0.001) and deoxycholylthreonine (*P* = 0.006) but significantly reduced deoxycholylcysteine (*P* = 0.002), compared to CON. Exploratory results that both RPG and RUG groups showed elevated relative abundances of 6-hydroxypentadecanedioic acid (*P* = 0.038) but reduced deoxycholylcysteine (*P* = 0.002), Pe(O-13:1/4:0) (*P* = 0.018), 4'-hydroxy-3'-methoxy-2-(2-piperidyl) acetophenone (*P* = 0.005), and Trp-Ser-Pro (*P* = 0.018) compared to CON. RPG group exhibited decreased relative abundance of pangamic acid (*P* = 0.033), 6-hydroxypentadecanedioic acid (*P* = 0.038), 5,6-dihydroxytetradecanedioic acid (*P* = 0.005), 3-(4-hydroxyphenyl)-lactate (*P* = 0.045), 5-hydroxyindole-3-acetic acid (*P* = 0.006), (2-oxo-2,3-dihydro-1H-indol-3-yl)-acetic acid (*P* = 0.038), and glutamic acid (*P* = 0.038) but increased alpha-amino-3-hydroxy-5-methyl-4-isoxazolepropionic acid versus RUG group. These metabolites were enriched in tryptophan metabolism, glutathione metabolism, adipocyte lipolysis regulation, serotonergic synapse, and bile acid synthesis pathways (Fig. S17). Correlation analysis indicated *Gordonibacter*, *Anaerobutyricum*, and *Butyrivibrio* were negatively correlated with DG (9D3/9D3/0:0) and cholylisoleucine but were positively correlated with chenodeoxycholylcysteine and deoxycholylcysteine (Fig. 10; *P* < 0.05).

**Table 10:** Comparison of relative abundance of key metabolites in jejunum between treatment groups

Item	Treatment^1^			SEM	*P*-value	*q*-value
	CON	RUG	RPG			
Cholylisoleucine	0.35^c^	2.53^a^	2.29^b^	0.249	0.005	0.023
Dg(9D3/9D3/0:0)	0.60^c^	3.02^a^	2.03^b^	0.178	0.001	0.015
Chenodeoxycholylcysteine	2.92^a^	1.34^b^	1.61^b^	0.256	0.006	0.064
Deoxycholylcysteine	3.49^a^	1.90^c^	2.79^b^	0.146	0.002	0.008
Pangamic acid	3.31^b^	4.17^a^	2.19^c^	0.463	0.033	> 0.100
Pe(O-13:1/4:0)	4.68^a^	4.10^b^	2.91^c^	0.341	0.018	0.083
Acetophenone, 4'-Hydroxy-3'-methoxy-2-(2-piperidyl)	4.07^a^	3.22^b^	3.33^b^	0.149	0.005	> 0.100
6-Hydroxypentadecanedioic acid	0.76^c^	2.67^a^	1.24^b^	0.393	0.038	0.099
Deoxycholylthreonine	3.49^c^	4.70^a^	4.12^b^	0.175	0.006	0.017
5,6-Dihydroxytetradecanedioic acid	1.20^b^	2.52^a^	0.80^c^	0.291	0.005	> 0.100
3-(4-Hydroxyphenyl)lactate	2.24^b^	3.30^a^	1.97^b^	0.298	0.045	0.094
5-Hydroxyindole-3-acetic acid	2.69^b^	3.82^a^	2.66^b^	0.207	0.006	0.052
Alpha-amino-3-hydroxy-5-methyl-4-isoxazolepropionic acid	2.54^b^	2.05^c^	2.97^a^	0.206	0.039	> 0.100
(2-Oxo-2,3-dihydro-1H-indol-3-Yl)acetic acid	1.46^b^	2.48^a^	1.66^b^	0.255	0.038	> 0.100
Cholylglutamine	4.22^b^	4.65^a^	4.38^b^	0.065	0.003	> 0.100
Trp-Ser-Pro	2.69^a^	1.93^b^	2.69^a^	0.167	0.018	0.097
Glutamic acid	2.08^b^	2.81^a^	2.00^b^	0.252	0.038	> 0.100

^a–c^Within a row, means with different superscript letters differ significantly (*P* < 0.05). Data are presented as least squares means with *n* = 6 per treatment

^1^CON: basal diet + 150 g/head/d palmitate coating; RUG: basal diet + 150 g/head/d palmitate coating + 150 g/head/d rumen-unprotected glucose; RPG: basal diet + 300 g/head/d rumen-protected glucose

## Discussion

### The rumen-centric pathway to intramuscular fat deposition

In this study, supplementation with RUG significantly enhanced DMI, chest girth, chest width, and abdominal girth compared to the CON group, indicating that rapidly fermentable energy in the rumen effectively promotes feed intake and physical development. The increased DMI may result from accelerated ruminal chyme movement due to rapid glucose fermentation, thereby alleviating feeding constraints [28]. Furthermore, elevated ruminal VFA concentrations may stimulate appetite-regulating hormones (e.g., LEP and GLP-1) modulating hypothalamic feeding centers through endocrine signaling to enhance feed intake [29]. Notably, supplementation with RUG exhibited significantly higher molar concentrations of total VFA, acetate, and propionate in rumen fluid compared to CON, directly evidencing the rapid and complete microbial fermentation of RUG in the rumen. In ruminants, acetate functions as the principal carbon source for de novo lipogenesis in peripheral tissues (primarily adipose), whereas propionate serves as the major gluconeogenic substrate in the liver and potentiates INS secretion through enteroendocrine signaling pathways [30]. In this study, supplementation with RUG demonstrated significantly elevated circulating INS, LEP, and cortisol levels. INS stimulates acetate utilization and upregulates lipogenic enzymes through coordinated activation of insulin receptor signaling pathways [31]. Thus, the altered ruminal fermentation pattern in the RUG group, characterized by concurrent increases in acetate and propionate concentrations, established complementary favorable conditions for adipogenesis through both substrate-driven metabolic pathways and hormone-mediated regulatory mechanisms [32, 33]. This rumen fermentation-driven enhancement of lipid synthesis was distinctly manifested in the slaughter performance and meat quality indices of the RUG group. Intramuscular fat content is a critical determinant of beef flavor, and tenderness, regulated by complex genetic and nutritional interactions [34]. Our findings demonstrate that supplying fermentable energy via the rumen pathway effectively promotes IMF deposition, aligning with practical observations that high-concentrate diets enhance beef marbling [35]. Elevated acetate concentrations provide abundant substrates for intramuscular adipocyte synthesis, while propionate-induced insulin hypersecretion likely amplifies the proliferation and differentiation capacity of intramuscular adipocyte precursors [6, 36]. Concurrently, reduced drip loss and shear force in RUG group beef further corroborate the positive role of elevated IMF in improving tenderness and water-holding capacity [37]. In this study, the RUG group demonstrated an activating effect on genes involved in fat synthesis in the longissimus dorsi muscle. The RUG group significantly up-regulated the gene expression of *ACC1*. ACC1 is the first rate-limiting enzyme in the de novo fatty acid synthesis pathway, responsible for catalyzing the conversion of acetyl-CoA to malonyl-CoA, thereby providing the core substrate for de novo fatty acid synthesis [38]. Moreover, the gene expression of *FABP4* was also significantly up-regulated. *FABP4* is primarily involved in fatty acid transport and intracellular metabolism, and its up-regulation can promote the transport of fatty acids into adipocytes and accelerate fat deposition [39]. The RUG group also showed an upward trend in the gene expression of *PPARγ*, a key transcription factor for lipid synthesis. *PPARγ* is recognized as the "master switch" for adipocyte differentiation and lipid generation, and its activation initiates the entire adipogenic program [40]. The coordinated up-regulation of this series of genes *(PPARγ*, *ACC1*, *FABP4*) collectively forms a complete and robust "pro-lipogenic signaling chain", promoting the rapid accumulation of intramuscular fat. This finding is closely aligned with the significantly increased intramuscular fat content and marbling score observed in the RUG group. Fatty acid composition analysis provided additional evidence for the "substrate-driven" lipogenic mechanism. RUG group meat samples exhibited significantly higher relative abundances of C14:0 and C15:0 in LT muscle compared to CON. The increased C14:0 levels indicate enhanced acetyl-CoA-dependent de novo lipogenesis [41], whereas elevated rumen microbiota-derived C15:0 (host-accumulated odd-chain FA) suggests altered microbial metabolic activity [42]_._ These collective findings demonstrate that metabolically available glucose in the rumen initiates a sequential regulatory cascade: enhanced production of volatile fatty acids promotes substrate availability for lipogenic processes, ultimately driving systemic lipid accumulation with preferential deposition in intramuscular adipocytes and visceral adipose tissues.

Rumen microbiota constitutes the central hub connecting dietary inputs with host metabolic outputs [43]. Although no significant effects in microbial alpha diversity and beta diversity were detected among groups in this study, potentially due to the uniformity of the basal diet formulation [44], the observed alterations in specific bacterial taxa abundances strongly suggest functional adaptive reorganization within the rumen microbial ecosystem. In the RUG group, the abundances of *norank_f_Clostridiaceae*, *Dongia*, *Olsenell*a, and *Pseudoflavonifractor* were significantly higher than in CON. Members of *Clostridiaceae* and *Olsenella* are critical fermenters of fibrous and non-fibrous carbohydrates, capable of producing substantial lactate and acetate, and their increased abundance may correlate with rapid glucose degradation [45]. While excessive lactate accumulation could theoretically induce ruminal acidosis, rumen pH remained stable in this trial, suggesting that generated lactate was likely rapidly converted to propionate by other microbes, thereby maintaining ruminal homeostasis [46]. *Pseudoflavonifractor* has also been reported to participate in carbohydrate fermentation and produce acetate and butyrate [47]. The enrichment of these acetate-producing genera aligns strongly with the significantly elevated acetate concentration in RUG group’s rumen fluid, confirming that structural changes in the microbiota drive fermentation pattern alterations [48]. Notably, *Dongia* abundance was significantly higher in RUG and RPG groups compared to CON. Correlation analyses revealed that *Dongia* abundance exhibited significant positive associations with DMI, chest girth, abdominal girth, backfat thickness, marbling score, and mesenteric fat weight. These correlations suggest that *Dongia* may play a pivotal role in energy metabolism and adipose deposition in cattle. As a recently identified member of *Clostridia*, *Dongia* may promote host growth and fat accumulation by efficiently utilizing glucose or its primary degradation products to generate VFA or other metabolites favorable for lipogenesis [49]. Additionally, *norank_f_Clostridiaceae* showed significant positive correlations with backfat thickness and omental fat weight, further reinforcing the role of these bacteria in driving adipogenesis. In contrast, RUG group significantly reduced the abundance of *Lactobacillus* and *Xylanivorax*. These genera have been proven to have special cellulose-decomposing capabilities in lignocellulose metabolism [50]. This corresponds to a microbial metabolic transformation, characterized by the inhibition of the degradation of recalcitrant fibers and the enhancement of the utilization of sugar fermentation substrates. PICRUSt2 functional prediction analysis revealed that the functional profile of the rumen microbial community was enriched in pathways related to secondary metabolite synthesis, amino acid biosynthesis, and carbon metabolism. Certain secondary metabolites produced by microorganisms may influence host physiological functions, such as modulating the proliferation of rumen epithelial cells, regulating fermentation patterns, affecting appetite, or participating in immune regulation [51]. The enrichment of amino acid biosynthesis pathways indicates that rumen microorganisms possess robust capabilities for ammonia assimilation and amino acid synthesis, which is of significant importance for maintaining ruminal nitrogen balance and improving dietary nitrogen utilization efficiency [52]. Carbon metabolism is directly linked to the production of volatile fatty acids and energy supply. The enrichment of carbon metabolism pathways suggests that the rumen microbial community exhibits active carbohydrate fermentation capacity [52]. Rumen metabolomics provided deeper insights into microbiota-driven metabolic reprogramming. Compared to CON, RUG group exhibited enrichment of metabolites in the propanoate metabolism pathway, with elevated levels of 3-hydroxypropanoic acid, which positively correlated with *Dongia* abundance. This aligns phenotypically with increased ruminal propionate concentrations. As an intermediate of propionate, hydroxypropionic acid can be transported into the bloodstream via glucose transporter type 2 (GLUT2) transporters in rumen epithelium, subsequently serving as a substrate for hepatic gluconeogenesis or direct delivery to jejunal epithelium [53]. Strikingly, metabolites were also enriched in the steroid biosynthesis pathway. Rumen microbiota synthesize diverse sterol compounds, and these microbial-derived steroids or their precursors may be absorbed by the host as signaling molecules or substrates, directly or indirectly modulating lipid metabolic networks [54]. This suggests that beyond VFAs, other microbiota-derived small molecules may play unrecognized roles in regulating adipose deposition via "rumen-host" crosstalk [26].

Emerging evidence indicates that the rumen and intestine form a multidimensional regulatory network via chyme flow, metabolite exchange, and microbial migration, triggering cascading metabolic responses [55, 56]. The modified ruminal fermentation profile in RUG group significantly increased jejunal acetate and propionate concentrations compared to other groups. This change coincided with selective enrichment of *Lachnotalea* in jejunal, a carbohydrate-fermenting genus known to generate acetate. Concurrently, the relative abundances of *Anaerobutyricum*, *Gordonibacter*, and *Adlercreutzia* decreased significantly in jejunum of the rumen unprotected glucose group. These bacterial genera have a significant positive correlation with the DCA and LCA levels in the jejunum. These coordinated microbial alterations suggest that dietary glucose supplementation may suppress colonization of bile acid-transforming bacteria, ultimately diminishing secondary bile acid biosynthesis [57].

### The jejunum-centric pathway to subcutaneous fat deposition

In stark contrast to RUG, cattle supplemented with RPG exhibited no changes in rumen fermentation parameters compared to the CON, yet demonstrated distinct patterns in growth performance, fat deposition, and nutrient digestibility. This conclusively demonstrates that RPG successfully bypassed ruminal fermentation and exerted its physiological functions in the small intestine, activating a "jejunum-centric pathway" fundamentally distinct from RUG. Notably, RPG group showed significantly higher EE digestibility than other groups, alongside superior NDF digestibility compared to RUG group. These advantages are attributable to two core mechanisms of RPG. First, by preventing excessive fermentable sugar influx into the rumen, the RUG group maintained relatively stable ruminal pH, favoring fibrolytic microbial activity and ensuring efficient utilization of dietary crude fiber-explaining its enhanced NDF digestibility [58]. Second, post-duodenal delivery of glucose stimulated secretion of intestinal hormones such as cholecystokinin (CCK) and secretin, which promoted bile and pancreatic fluid release [59]. This aligns with the observed significant increase in LCA levels in RPG group. Consequently, RPG likely optimized dietary EE hydrolysis, and absorption through enhanced digestive fluid secretion, ultimately elevating EE digestibility [60, 61]. This improved digestibility enabled RPG cattle to extract more net energy per kilogram of feed, providing a highly efficient energy foundation for growth and fat deposition, which partially explains why RPG group achieved body size gains and backfat thickness comparable to RUG group despite lower DMI.

This study further revealed significant upregulation of key adipogenic genes in subcutaneous adipose tissue of the RPG group. Compared to other groups, RPG group exhibited activation of multiple critical nodes in the lipid synthesis and regulatory network. *ACC1*, the rate-limiting enzyme for de novo fatty acid synthesis, demonstrated upregulated expression directly indicative of enhanced lipogenic capacity [38]. *PPARγ* and *SREBP*-*1c*, renowned as "master regulators" of adipocyte differentiation and lipogenesis, play pivotal roles in adipose tissue remodeling and fatty acid cycling, essential for maintaining adipocyte morphology and function [62]. The collective upregulation of these genes signifies a transition in the subcutaneous adipose tissue of the RPG group into a hyperactive "synthesis-remodeling" state. The transcriptional activation likely originates from intestinal glucose absorption. Following jejunal uptake of RPG, elevated peripheral blood glucose levels strongly stimulated INS secretion from pancreatic β-cells. The INS signaling pathway (via PI3K-Akt) phosphorylates and activates transcription factor Fox1, thereby alleviating its suppression of *SREBP-1c* transcription. Concurrently, the Akt pathway directly or indirectly promotes *SREBP-1c* processing and activation [63, 64]. Activated *SREBP-1c* subsequently translocates to the nucleus, binding to and upregulating lipogenic genes (*PPARγ*, *ACC1*) [65]. Notably, this study identified coordinated upregulation of *TGR5* in subcutaneous adipose and jejunal tissues with core lipogenic genes (*PPARγ*, *SREBP-1c*) in the RPG group, suggesting a novel signaling cascade: RPG activates jejunal *TGR5* signaling, inducing secretion of INS, GLP-1, and 5-HT, which amplify the activation of master regulators (*PPARγ*, *SREBP-1c*), ultimately driving systemic lipogenic gene networks [66, 67]. This establishes a precise signaling axis from the "rumen-jejunum axis" to transcriptional regulation in distal adipose tissue, contrasting sharply with "substrate-driven" reliance on rumen fermentation products in the RUG group [68, 69]. The RPG group represents a "signal-driven" transcriptional regulatory mechanism highlighting divergent evolutionary strategies for adipose deposition.

Compared with CON, the RPG group exhibited significantly increased relative abundance of *norank_f_Clostridiaceae* in the jejunum, alongside marked reductions in *Gordonibacter* and *Adlercreutzia*. Concurrently, the RPG group demonstrated substantially lower jejunal DCA content than CON, suggesting that RPG supplementation may also inhibit colonization of bile acid-transforming bacteria in the jejunum, thereby reducing DCA accumulation. The altered ruminal microbiota in RPG group indicated an indirect regulatory paradigm. Although RPG was originally designed to deliver glucose directly to the small intestine [56], ruminal microbial changes in RPG group likely originated not from direct glucose utilization but rather from systemic physiological adaptations (e.g., INS, GLP-1, 5-HT) following intestinal glucose absorption, which subsequently modified the ruminal microenvironment through feedback mechanisms [70]. Studies have shown that insulin, GLP-1, and 5-HT can stimulate the proliferation of rumen epithelial cells, change the rumen peristalsis, permeability, and availability of nutrients, thereby indirectly regulating microbial community structure [70]. RPG group displayed significant increases in *Clostridium*, *norank_f_Clostridiaceae*, and *Dongia* in the rumen. The *Dongia* and *norank_f_Clostridiaceae* were identified as group-specific biomarkers via LEfSe analysis. This demonstrates that host physiological state alterations can selectively enrich functionally specialized microbial taxa even in the absence of direct substrate provision [71, 72]. Differentially abundant ruminal metabolites in RPG group were predominantly enriched in host-centric signaling pathways, including MAPK signaling, growth hormone synthesis/secretion, and NF-κB signaling, rather than microbial primary metabolic pathways [73]. These findings collectively validate that ruminal metabolic changes induced by RPG are indirectly mediated through host physiological regulation. Following efficient jejunal absorption of RPG, INS, EPI, LEP, GLP-1, 5-HT, and T4 secretion were stimulated. These hormones systemically modulated ruminal epithelial functions via MAPK and NF-κB pathway activation, ultimately reducing ruminal serotonin and 5-hydroxyindoleacetaldehyde levels. A critical commonality across groups involved significant perturbations in the tryptophan metabolism pathway. Both RPG and RUG groups exhibited lower 3-methyloxindole abundance compared to CON. As tryptophan serves as a co-metabolized substrate for both microbiota and host, its derivatives (indole compounds and serotonin) play pivotal roles in gut motility and energy homeostasis regulation [74]. The attenuated levels of these metabolites suggest that glucose supplementation (RPG or RUG) reprograms microbial tryptophan utilization patterns, potentially influencing feeding behavior and energy partitioning through gut-brain axis modulation [75]. Intriguingly, this "reverse" regulatory axis from intestine to rumen provides compelling evidence for bidirectional communication within the rumen-jejunum axis.

### Differential fat deposition patterns and implications of two glucose supplementation strategies

**Fig. 11:**
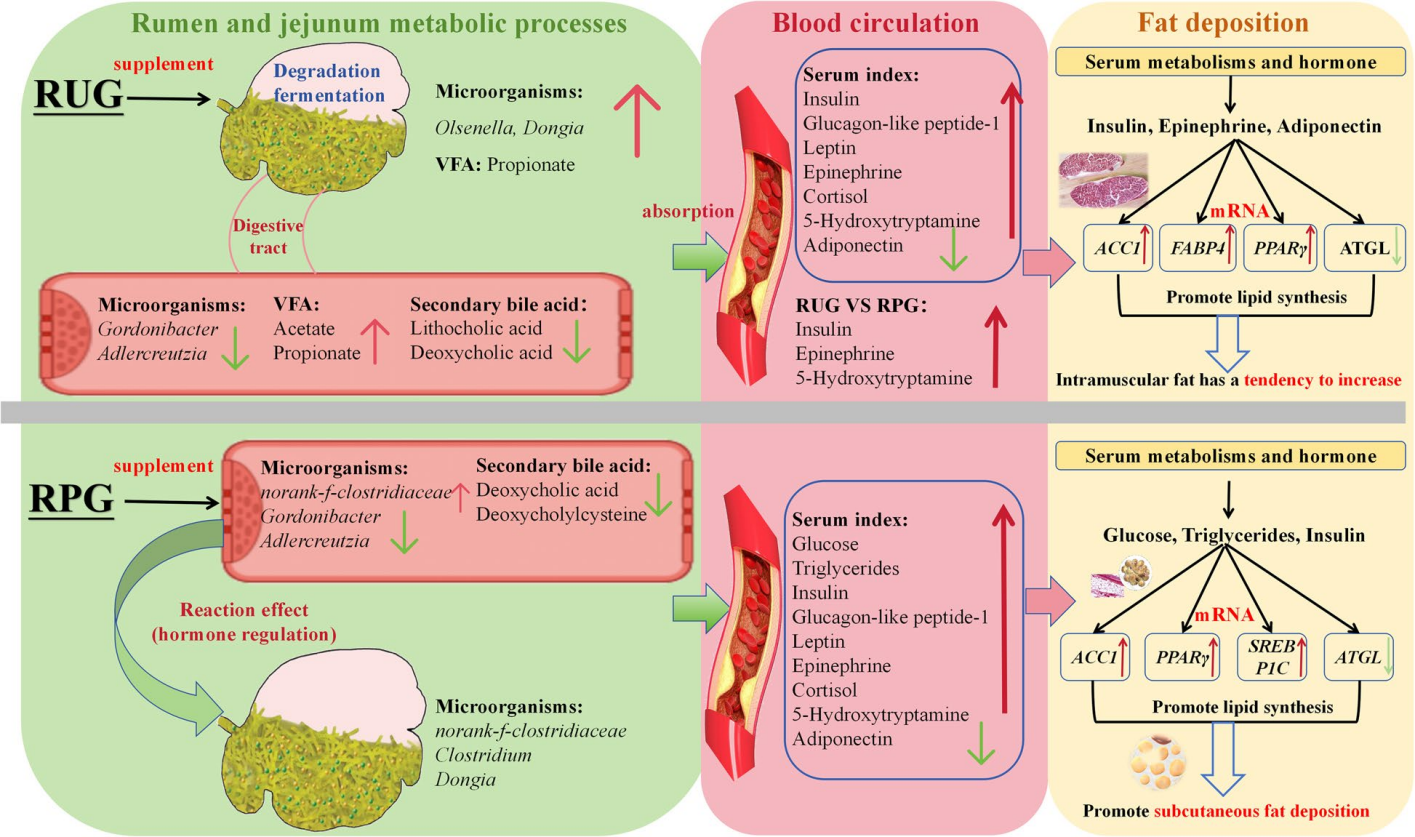
Differential regulation mechanism of dietary supplementation of RUG and RPG on lipid deposition. RUG: Rumen-unprotected glucose, RPG: Rumen-protected glucose

Comparative analysis of fat deposition patterns between RPG and RUG groups revealed distinct preferential effects on specific fat depots (Fig. 11). The RUG group demonstrated pronounced increases in omental fat weight compared with CON, with particularly marked elevation in mesenteric fat weight and marbling score compared to RPG group. While the RPG group also exhibited increased backfat thickness and omental fat weight compared with CON, its stimulatory effects on mesenteric and IMF deposition were attenuated relative to the RUG group. This depot-specific "lipid repurposing" phenomenon may originate from fundamental mechanistic differences between the two pathways. The "substrate-driven" mechanism in RPG group generated substantial VFA and microbial metabolites entering systemic circulation via the portal vein. Visceral adipose depots (e.g., mesenteric and omental fat), being the first fat reservoirs encountered in portal circulation, preferentially sequester and utilize these abundant lipogenic precursors, leading to their rapid expansion [76]. Intramuscular adipocytes, embedded within muscle fibers, exhibit metabolic activity highly dependent on circulating substrate concentrations. Sustained elevation of systemic acetate and propionate levels in the RUG group consequently enhanced intramuscular lipogenesis, ultimately manifesting as superior marbling development [37]. In contrast, the signaling transduction mechanism observed in the RPG group was predominantly dependent on systemic hormonal regulation, particularly involving INS and GLP-1 circulatory pathways. While these systemic signals act broadly on adipose tissues, depot-specific differences in adipocyte sensitivity likely exist. Subcutaneous adipose tissue serves as the primary energy buffer during caloric surplus, exhibiting greater proliferative and hypertrophic potential [77]. This depot demonstrated heightened responsiveness to anabolic signals like INS, consistent with our observation of significantly upregulated lipogenic gene expression in subcutaneous fat. In contrast, intramuscular adipogenesis requires coordinated substrate availability and microenvironmental signaling—conditions not sufficiently provided by transcriptional activation alone [36], accounting for its attenuated development in the RPG group compared to the RUG group. These differential effects hold critical practical implications for manipulation of adipose deposition patterns in beef cattle production. For beef production prioritizing marbling quality, strategic supplementation of rumen-fermentable carbohydrates (e.g., RUG or high-starch grains) during late finishing phases represents an effective approach. Conversely, when optimizing feed efficiency while enhancing subcutaneous fat coverage for carcass grading and controlling visceral fat deposition, RPG supplementation may prove advantageous.

## Conclusions

Dietary supplementation with RUG tends to promote intramuscular fat deposition through dual mechanisms: rapid generation of acetate and propionate as lipid precursors, coupled with microbial restructuring (notable enrichment of *Clostridiaceae* and *Dongia*) and metabolic reprogramming (activation of steroid biosynthesis pathway with reduced DCA and LCA concentrations) via the rumen-jejunum axis. RPG enhances energy utilization efficiency through improved EE and NDF digestibility, while activating a "jejunum absorption-hormonal signaling-distal tissue response" cascade that specifically upregulates the *PPARγ/SREBP1c*-centered lipogenic network in subcutaneous adipose tissue. Collectively, these findings not only clarify the divergent effects and mechanisms of two glucose supplementation strategies in beef production but also advance our understanding of how the "rumen-jejunum axis" integrates nutritional signals to differentially regulate fat depots.

## Supplementary Information

Additional file 1: Table S1 Primer sequences. Fig. S1 Effects of dietary supplementation of rumen-protected and unprotected glucose on meat color and pH of Xinjiang Brown cattle. Fig. S2 Effects of dietary supplementation of rumen-protected and unprotected glucose on the sequencing depth of rumen microorganisms in Xinjiang Brown cattle. A Sobs index. B Coverage index. Fig. S3 The results of the differences in the number of operational taxonomic unitsof rumen fluid between treatment groups. The treatment groups included CON, RUG or RPG. The number within each differently colored overlapping area is the number of OTUs shared by the overlapping groups. Nonoverlapping areas indicate the number of OTUs unique to each group. Fig. S4 Effects of dietary supplementation of rumen-protected and unprotected glucose on the alpha diversity of rumen microorganisms in Xinjiang Brown cattle. A ACE index. B Chao index. C Shannon index. D Coverage index. E Sobs index. F Simpson index. Fig. S5 Effects of dietary supplementation of rumen-protected and unprotected glucose on the beta diversity of rumen microorganisms in Xinjiang Brown cattle. A PCoaA on OTU level. B NMDS on OTU level. Fig. S6 The differences in the relative abundance of bacteria between treatment groups in rumen fluid samples of Xinjiang Brown cattle. A Relative abundances of the top 15 bacterial taxa at the phylum level. B Relative abundances of the top 30 bacterial taxa at the genus level. Fig. S7 The significantly differential microorganisms based on the linear discriminant analysis effect sizecladogram in rumen fluid samples of Xinjiang Brown cattle among treatment groups. Fig. S8 The difference of metabolites in rumen fluid samples of Xinjiang Brown cattle among treatment groups. A The amount of data of up-regulated and down-regulated differential metabolites between CON and RUG. B The amount of data of up-regulated and down-regulated differential metabolites between RUG and RPG. C The amount of data of up-regulated and down-regulated differential metabolites between CON and RPG. Fig. S9 The differential pathways enriched by Key metabolitesensembles in rumen fluid samples of Xinjiang Brown cattle. Fig. S10 Effects of dietary supplementation of rumen-protected and unprotected glucose on the sequencing depth of jejunum microorganisms in Xinjiang Brown cattle. A Sobs index. B Coverage index. Fig. S11 The results of the differences in the number of operational taxonomic unitsof jejunum fluid between treatment groups.The treatment groups included CON, RUGB or RPG. The number within each differently colored overlapping area is the number of OTUs shared by the overlapping groups. Nonoverlapping areas indicate the number of OTUs unique to each group. Fig. S12 Effects of dietary supplementation of rumen-protected and unprotected glucose on the alpha diversity of jejunum microorganisms in Xinjiang Brown cattle. A ACE index. B Chao index. C Shannon index. D Coverage index. E Simpson index. Fig. S13 Effects of dietary supplementation of rumen-protected and unprotected glucose on the beta diversity of jejunum microorganisms in Xinjiang Brown cattle. A PCoaA on OTU level. B NMDS on OTU level. Fig. S14 The differences in the relative abundance of bacteria between treatment groups in jejunum fluid samples of Xinjiang Brown cattle. A Relative abundances of the top 15 bacterial taxa at the phylum level. B Relative abundances of the top 30 bacterial taxa at the genus level. Fig. S15 The significantly differential microorganisms based on the linear discriminant analysis effect sizecladogra in jejunum fluid samples of Xinjiang Brown cattle among treatment groups. Fig. S16 The difference of metabolites in jejunum fluid samples of Xinjiang Brown cattle among treatment groups. A The amount of data of up-regulated and down-regulated differential metabolites between CON and RUG. B The amount of data of up-regulated and down-regulated differential metabolites between RUG and RPG. C The amount of data of up-regulated and down-regulated differential metabolites between CON and RPG. Fig. S17 The differential pathways enriched by Key metabolitesensembles in jejunum fluid samples of Xinjiang Brown cattle

## Data Availability

The datasets used and/or analyzed during the current experiment are available from the corresponding author on reasonable request. Raw sequencing data of all 16S rRNA sequences have been deposited into the NCBI Sequence Read Archive (SRA) under accession numbers PRJNA1440057. The metabolomics data have been deposited to the EMBL-EBI MetaboLights database with the identifier MTBLS14139.

## Abbreviations

5-HT: 5-Hydroxytryptamine
ADP: Adiponectin
ADG: Average daily gain
BW: Body weight
CHO: Cholesterol
DCA: Deoxycholic acid
DMI: Dry matter intake
EPI: Epinephrine
FCR: Feed conversion ratio
GLU: Glucose
GLP-1: Glucagon-like peptide-1
INS: Insulin
LCA: Lithocholic acid
LEP: Leptin
LT: Longissimus thoracis
MCP: Microbial crude protein
NH_3_-N: Ammonia nitrogen
RPG: Rumen protected glucose
RUG: Rumen unprotected glucose
SCFA: Short-chain fatty acid
T3: Triiodothyronine
T4: Thyroxine
TG: Triglycerides
TMR: Total mixed ration
VFA: Volatile fatty acid
