# Supplementary material for: Rumen unprotected glucose tends to promote intramuscular fat deposition via microbial volatile fatty acid-mediated metabolic reprogramming in cattle: a multi-omics perspective through the rumen-jejunum axis
Source: J Anim Sci Biotechnol. 2026 May 25;17:99. doi: 10.1186/s40104-026-01419-6 (PMC13200308; doi:10.1186/s40104-026-01419-6)
Supplement: Supplementary file 1 — Additional file 1: Table S1 Primer sequences. Fig. S1 Effects of dietary supplementation of rumen-protected and unprotected glucose on meat color and pH of Xinjiang Brown cattle. Fig. S2 Effects of dietary supplementation of rumen-protected and unprotected glucose on the sequencing depth of rumen microorganisms in Xinjiang Brown cattle. A Sobs index. B Coverage index. Fig. S3 The results of the differences in the number of operational taxonomic unitsof rumen fluid between treatment groups. The treatment groups included CON, RUG or RPG. The number within each differently colored overlapping area is the number of OTUs shared by the overlapping groups. Nonoverlapping areas indicate the number of OTUs unique to each group. Fig. S4 Effects of dietary supplementation of rumen-protected and unprotected glucose on the alpha diversity of rumen microorganisms in Xinjiang Brown cattle. A ACE index. B Chao index. C Shannon index. D Coverage index. E Sobs index. F Simpson index. Fig. S5 Effects of dietary supplementation of rumen-protected and unprotected glucose on the beta diversity of rumen microorganisms in Xinjiang Brown cattle. A PCoaA on OTU level. B NMDS on OTU level. Fig. S6 The differences in the relative abundance of bacteria between treatment groups in rumen fluid samples of Xinjiang Brown cattle. A Relative abundances of the top 15 bacterial taxa at the phylum level. B Relative abundances of the top 30 bacterial taxa at the genus level. Fig. S7 The significantly differential microorganisms based on the linear discriminant analysis effect sizecladogram in rumen fluid samples of Xinjiang Brown cattle among treatment groups. Fig. S8 The difference of metabolites in rumen fluid samples of Xinjiang Brown cattle among treatment groups. A The amount of data of up-regulated and down-regulated differential metabolites between CON and RUG. B The amount of data of up-regulated and down-regulated differential metabolites between RUG and RPG. C The amount of data of up-regulated [file 40104_2026_1419_MOESM1_ESM.docx]

Table S1 Primer sequences

| Primer name | Sequence (5' to 3') |
| --- | --- |
| FXR-F | TCTGTGGAGACCGAGCATCTGG |
| FXR-R | TCTTGGCACTTCCTTCGCATGTAC |
| PPARγ-F | GACGACAGACAAATCACCGT |
| PPARγ-R | CAGGGGACTGATGTGCTTGA |
| SREBP-1C-F | CTGACCGACATAGAAGACATGC |
| SREBP-1C-R | CGTAGGGCGGGTCGAATAG |
| ACC1-F | CTGGAGGTTTTTGCCTCCCA |
| ACC1-R | TCCACTTCCAAAAAGAACTCAGAGA |
| ATGL-F | GGAGCTTATCCAGGCCAATGT |
| ATGL-R | TCATAGAGCGGCAGGTTGTC |
| HSL-F | TGGGAGGGCCTCACGG |
| HSL-R | AAGGCCATGTTGTCCTCTGC |
| CPT1-F | CCAGCTCAGAGACAAATGCCC |
| CPT1-R | TGGCATCTCTCCAGCCCTTAG |
| TGR5-F | GGACAACTCCCTGACACTCG |
| TGR5-R | GGCATGCATGACTGTAGGT |
| β-actin F | GCGGCATTCACGAAACTACC |
| β-actin R | TCTTCATTGTGCTGGGTGCC |

**Supplementary Figure**


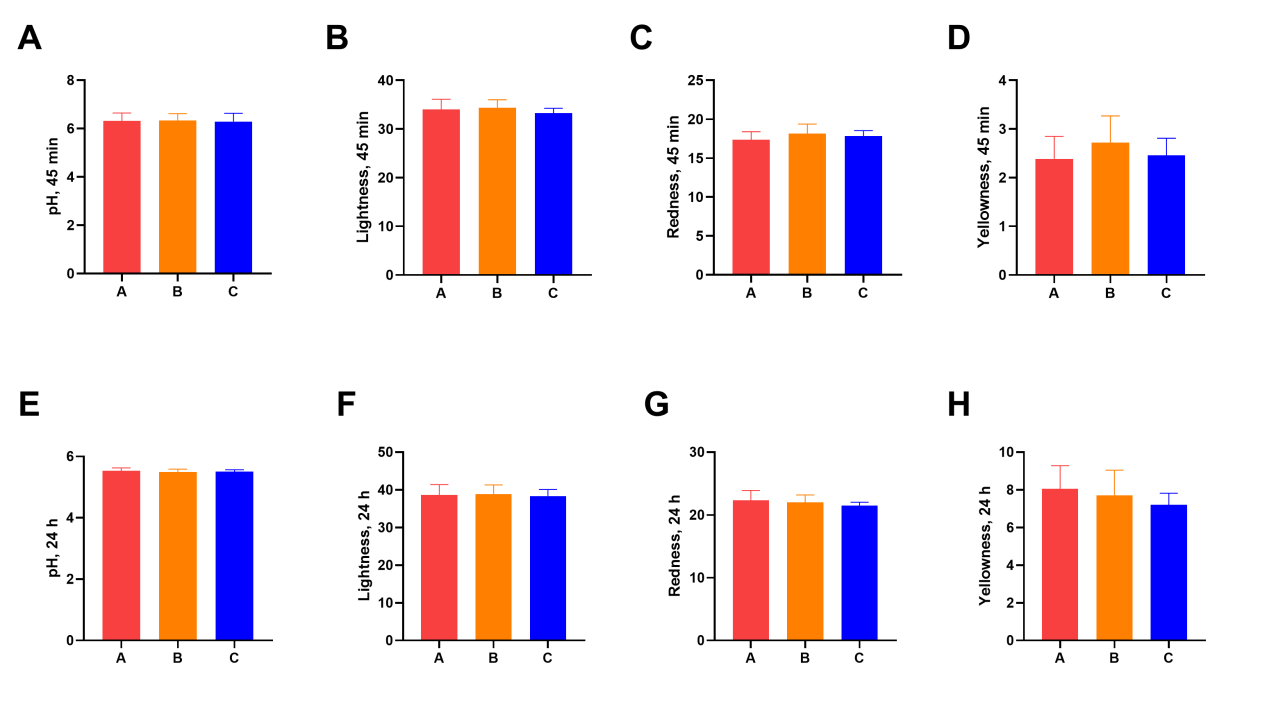


**Fig. S1** Effects of dietary supplementation of rumen-protected and unprotected glucose on meat color and pH of Xinjiang Brown cattle.


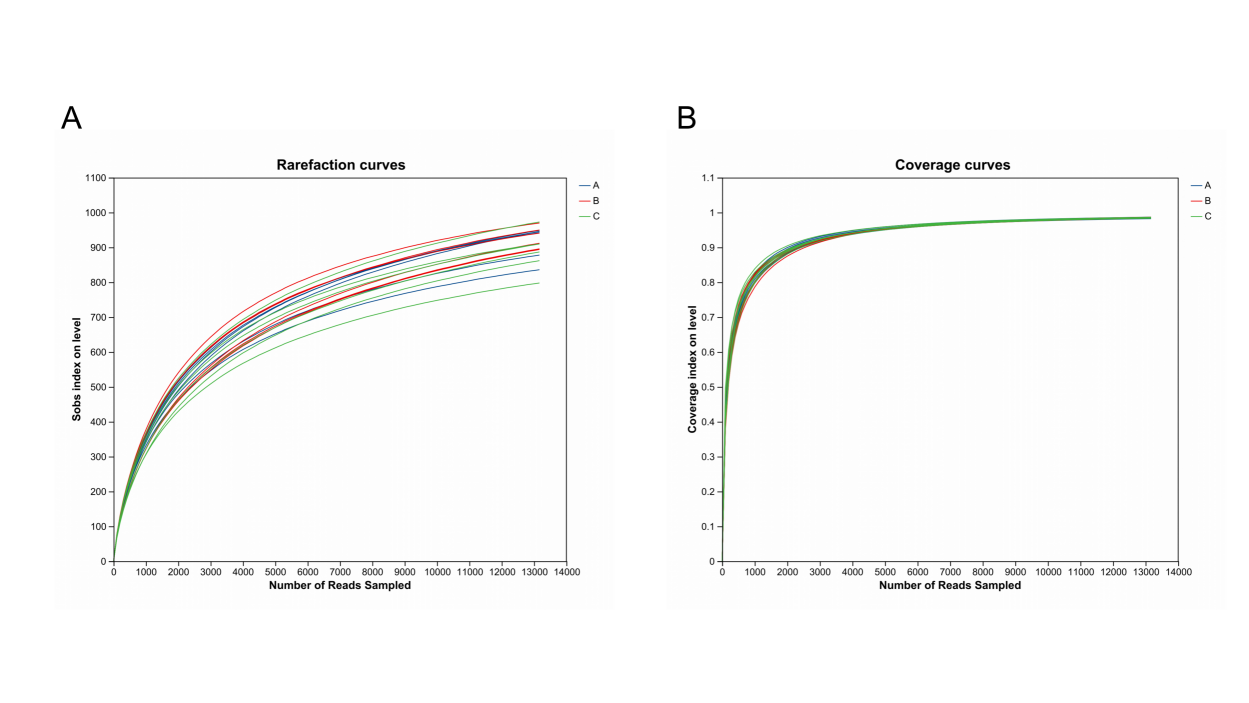


**Fig. S2** Effects of dietary supplementation of rumen-protected and unprotected glucose on the sequencing depth of rumen microorganisms in Xinjiang Brown cattle. **A** Sobs index. **B** Coverage index.


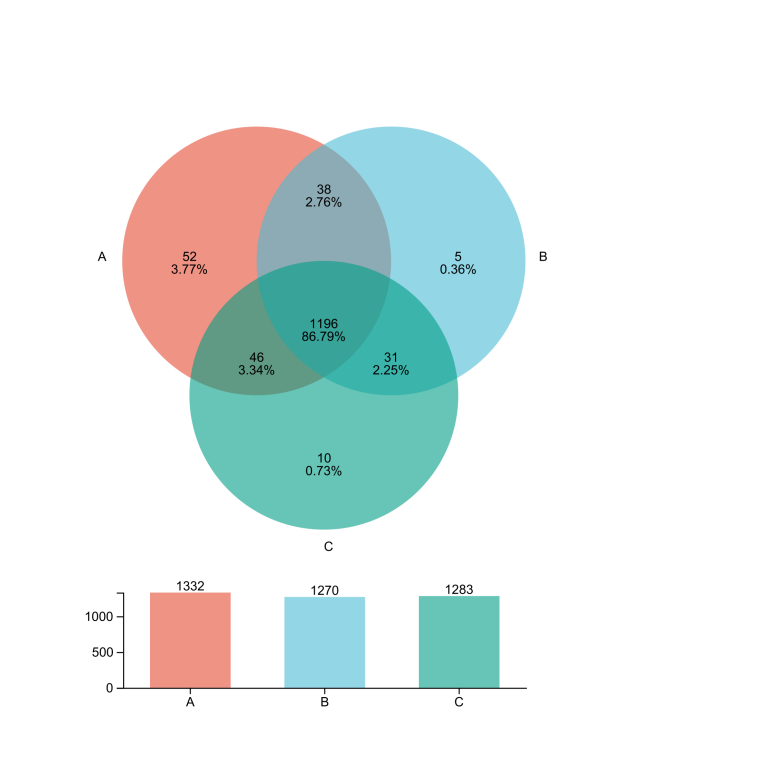


**Fig. S3** The results of the differences in the number of operational taxonomic units (OTUs) of rumen fluid between treatment groups. The treatment groups included Group A (control), Group B (rumen-unprotected glucose) or Group C (rumen-protected glucose). The number within each differently colored overlapping area is the number of OTUs shared by the overlapping groups. Nonoverlapping areas indicate the number of OTUs unique to each group.


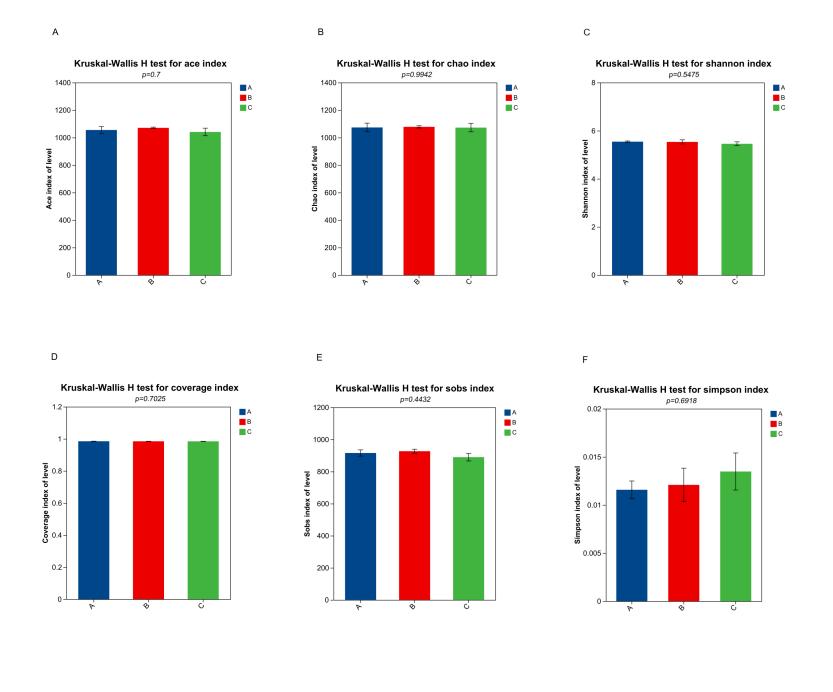


**Fig. S4** Effects of dietary supplementation of rumen-protected and unprotected glucose on the ɑ-diversity of rumen microorganisms in Xinjiang Brown cattle. **A** ACE index. **B** Chao index. **C** Shannon index. **D** Coverage index. **E** Sobs index. **F** Simpson index.


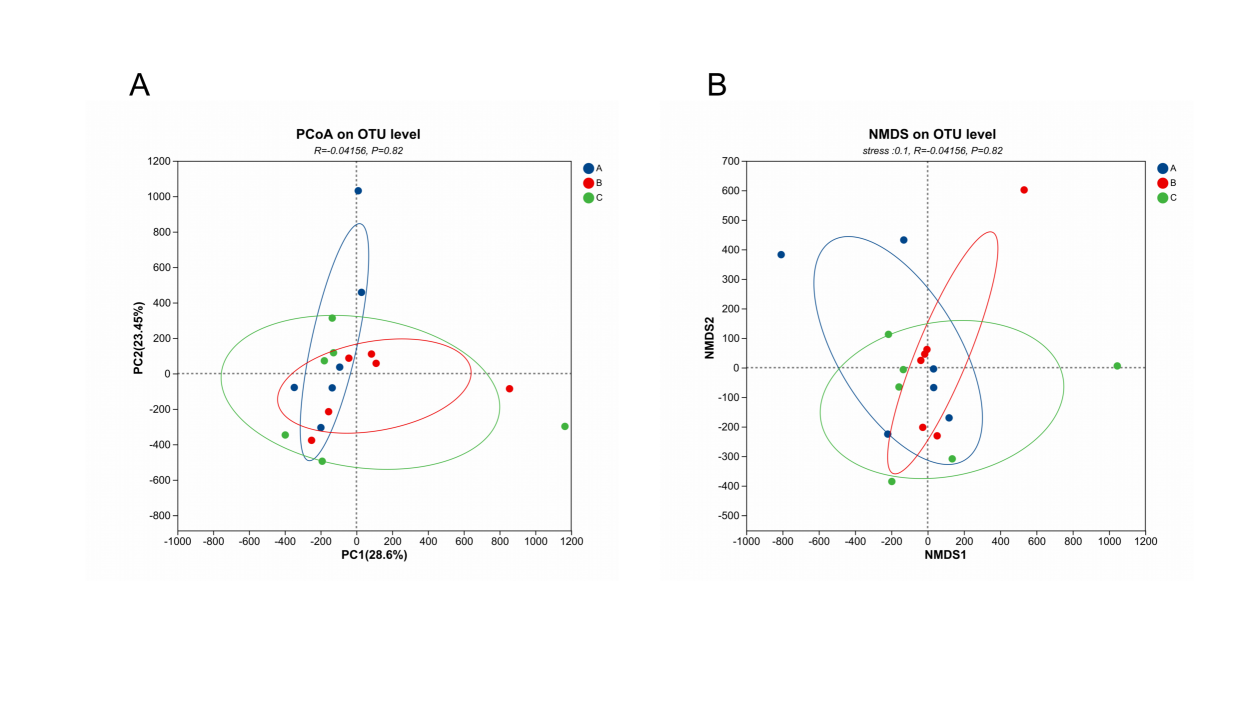


**Fig. S5** Effects of dietary supplementation of rumen-protected and unprotected glucose on the β-diversity of rumen microorganisms in Xinjiang Brown cattle. **A** PCoaA on OTU level. **B** NMDS on OTU level.


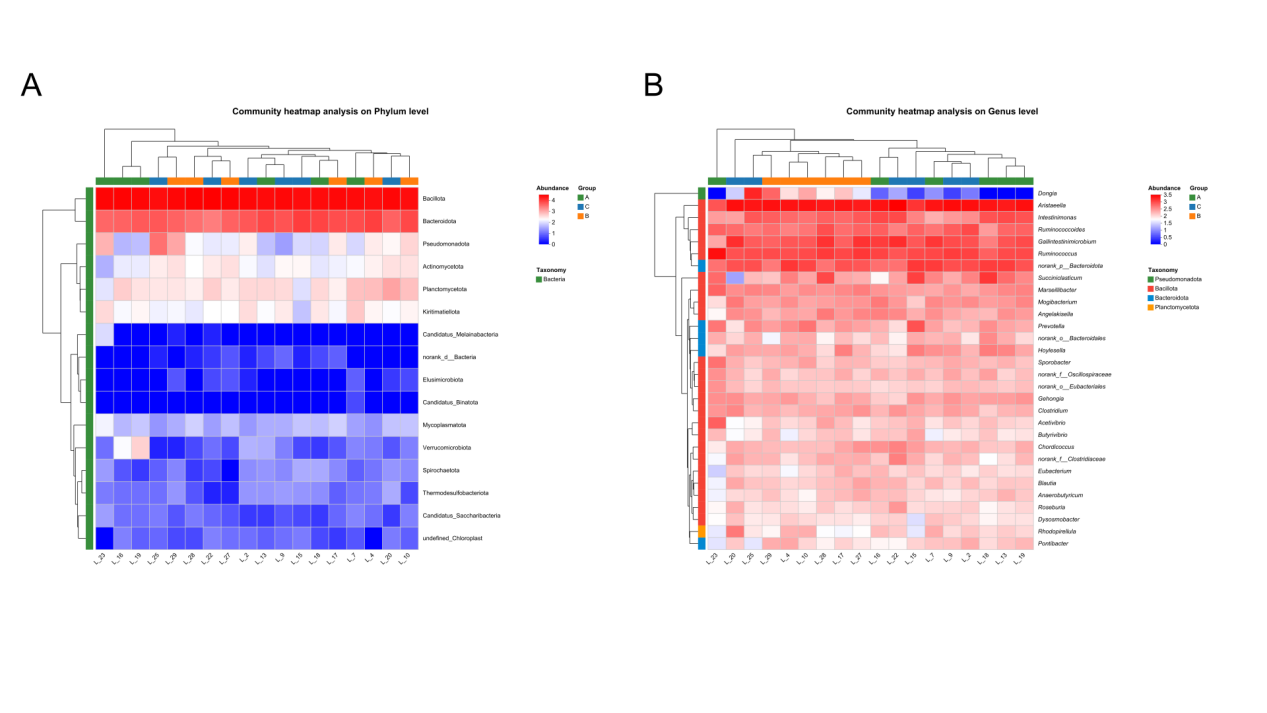


**Fig. S6** The differences in the relative abundance of bacteria between treatment groups in rumen fluid samples of Xinjiang Brown cattle. **A** Relative abundances of the top 15 bacterial taxa at the phylum level. **B** Relative abundances of the top 30 bacterial taxa at the genus level


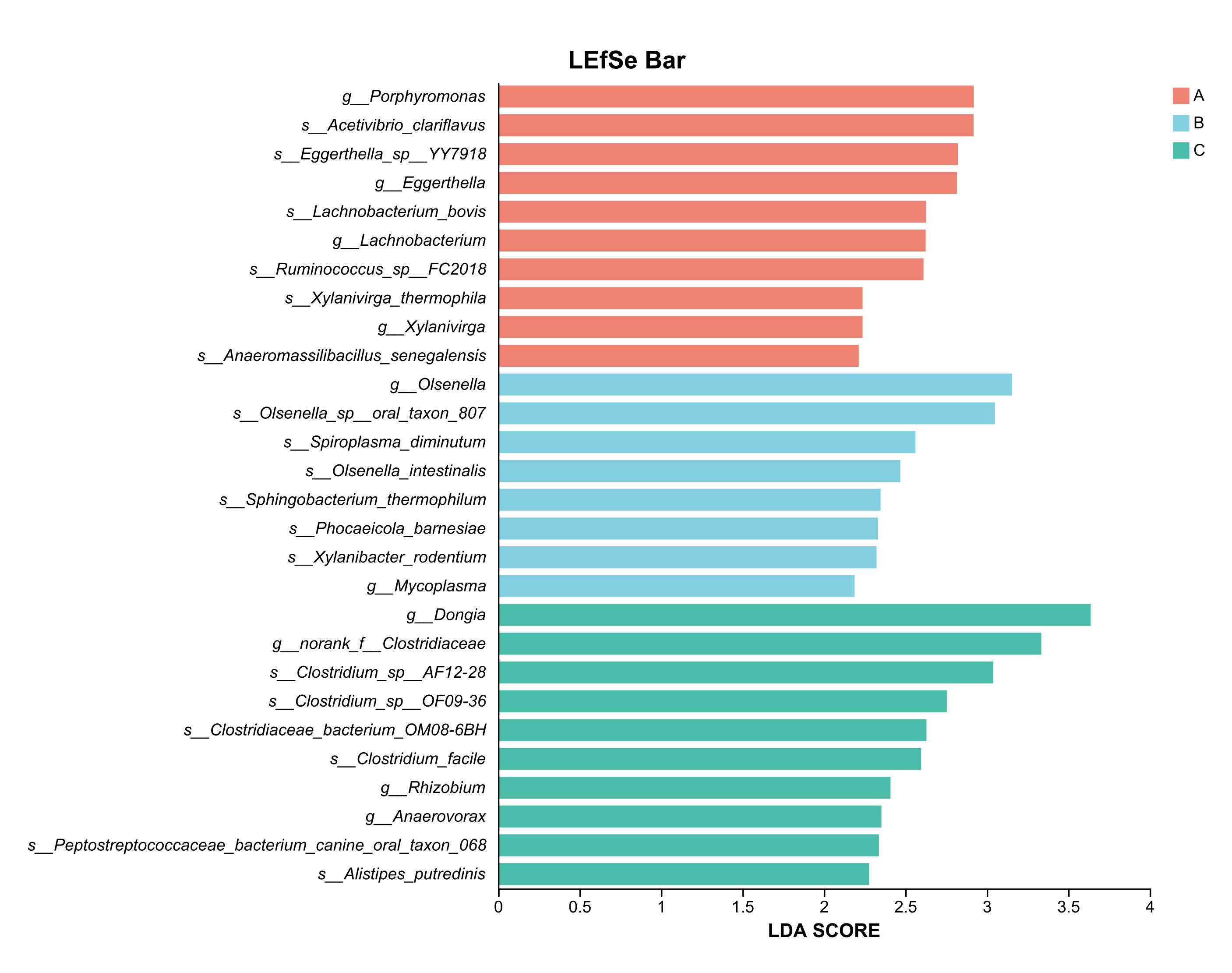


**Fig. S7** The significantly differential microorganisms based on the linear discriminant analysis effect size (LEfSe) cladogra in rumen fluid samples of Xinjiang Brown cattle among treatment groups.


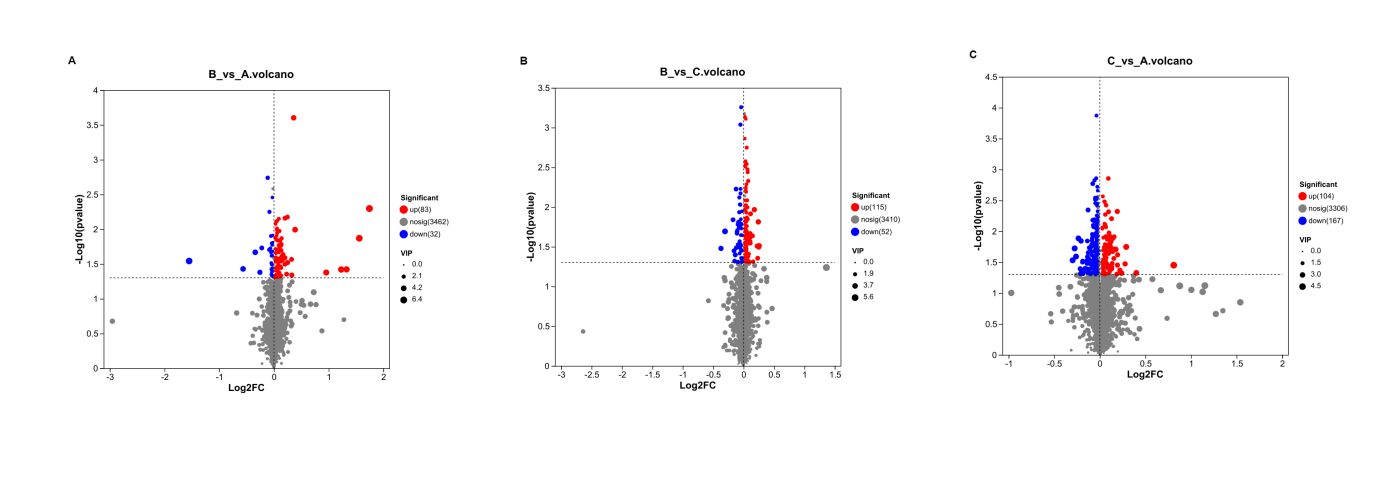


**Fig. S8** The difference of metabolites in rumen fluid samples of Xinjiang Brown cattle among treatment groups. **A** The amount of data of up-regulated and down-regulated differential metabolites between group A and group B. **B** The amount of data of up-regulated and down-regulated differential metabolites between group B and group C. **C** The amount of data of up-regulated and down-regulated differential metabolites between group A and group C.


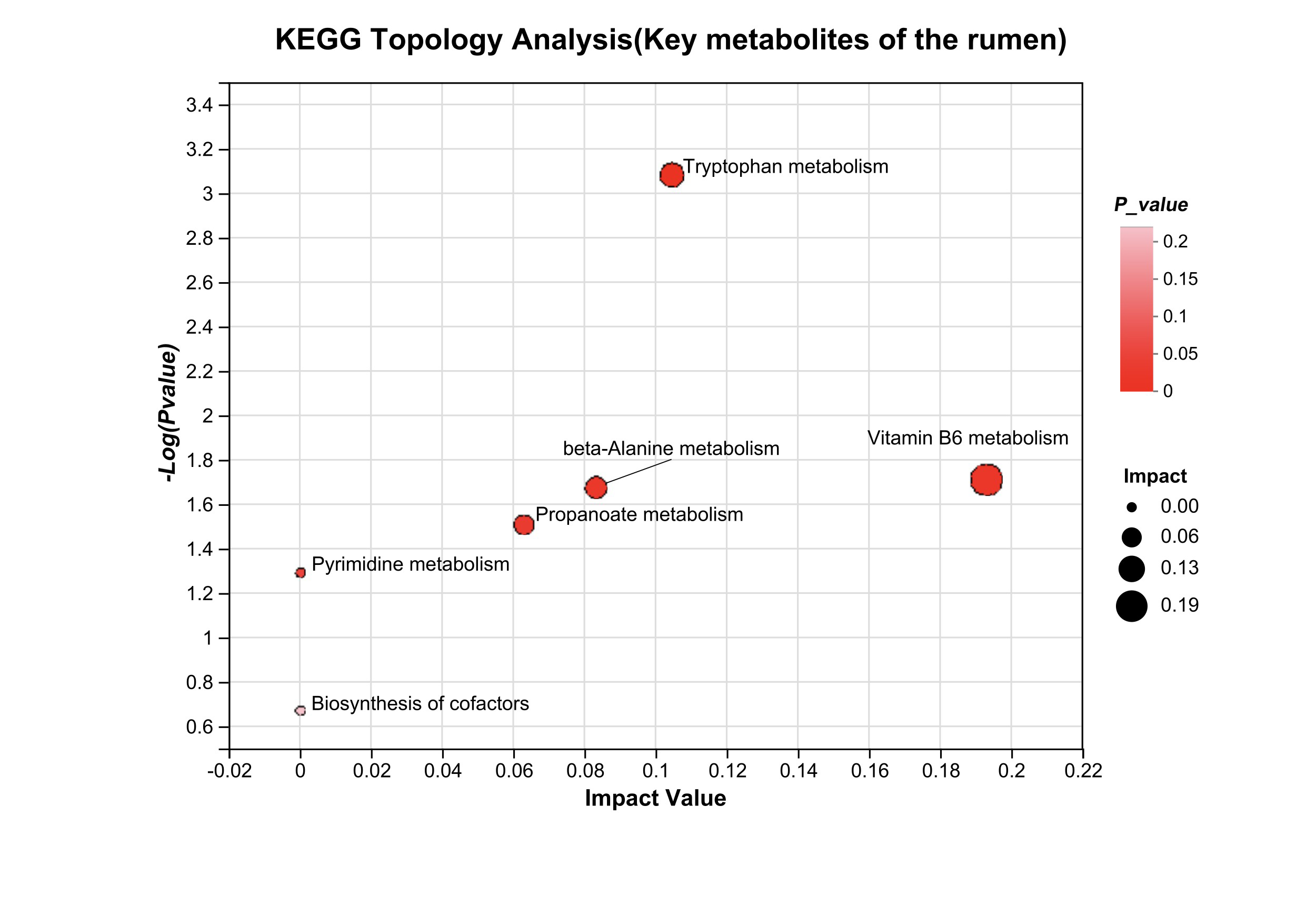


**Fig. S9** The differential pathways enriched by Key metabolites (13) ensembles in rumen fluid samples of Xinjiang Brown cattle.


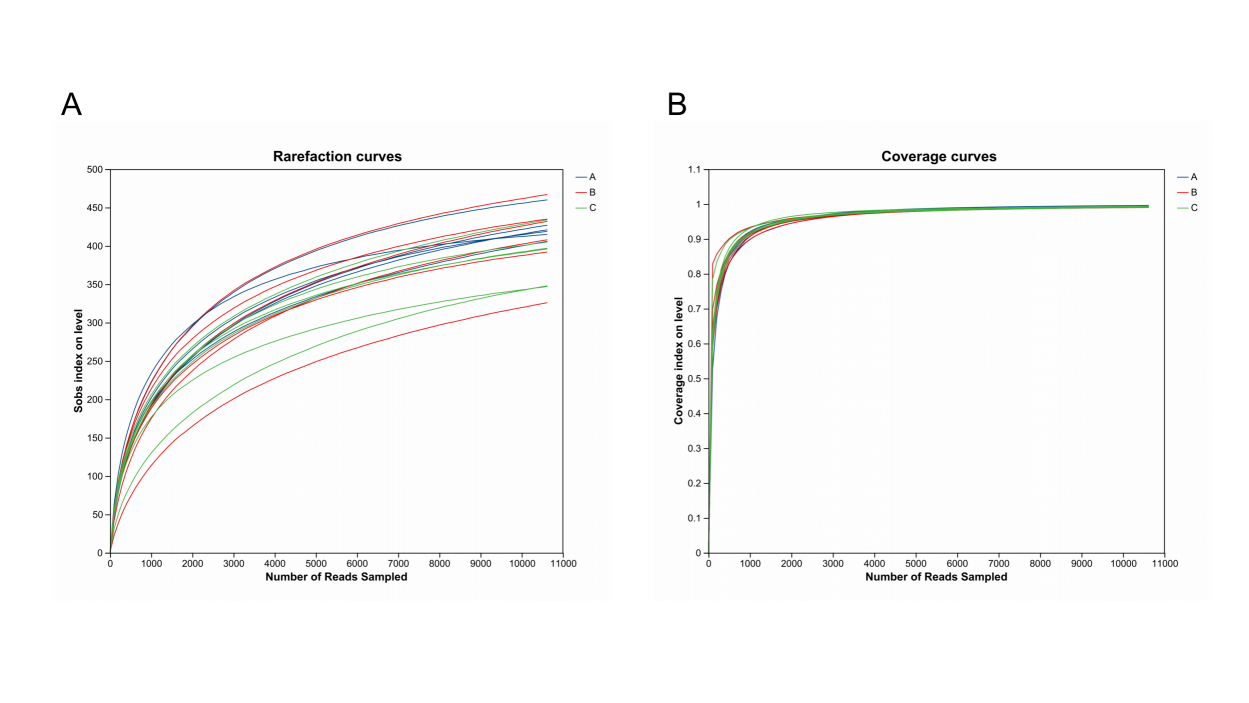


**Fig. S10** Effects of dietary supplementation of rumen-protected and unprotected glucose on the sequencing depth of jejunum microorganisms in Xinjiang Brown cattle. **A** Sobs index. **B** Coverage index.


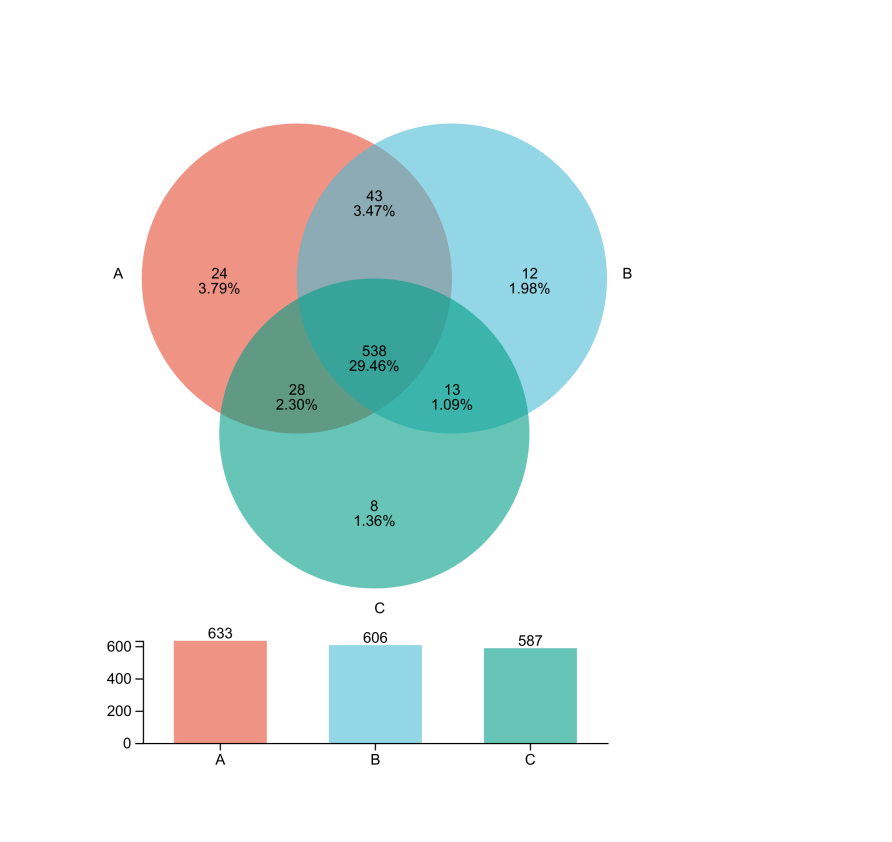


**Fig. S11** The results of the differences in the number of operational taxonomic units (OTUs) of jejunum fluid between treatment groups.The treatment groups included Group A (control), Group B (rumen-unprotected glucose) or Group C (rumen-protected glucose). The number within each differently colored overlapping area is the number of OTUs shared by the overlapping groups. Nonoverlapping areas indicate the number of OTUs unique to each group.


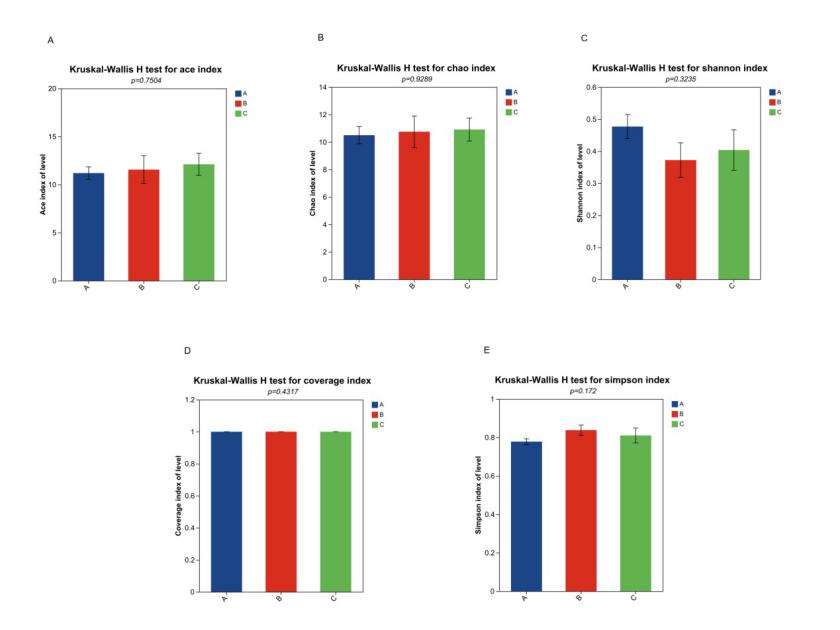


**Fig. S12** Effects of dietary supplementation of rumen-protected and unprotected glucose on the ɑ-diversity of jejunum microorganisms in Xinjiang Brown cattle. **A** ACE index. **B** Chao index. **C** Shannon index. **D** Coverage index. **E** Simpson index.


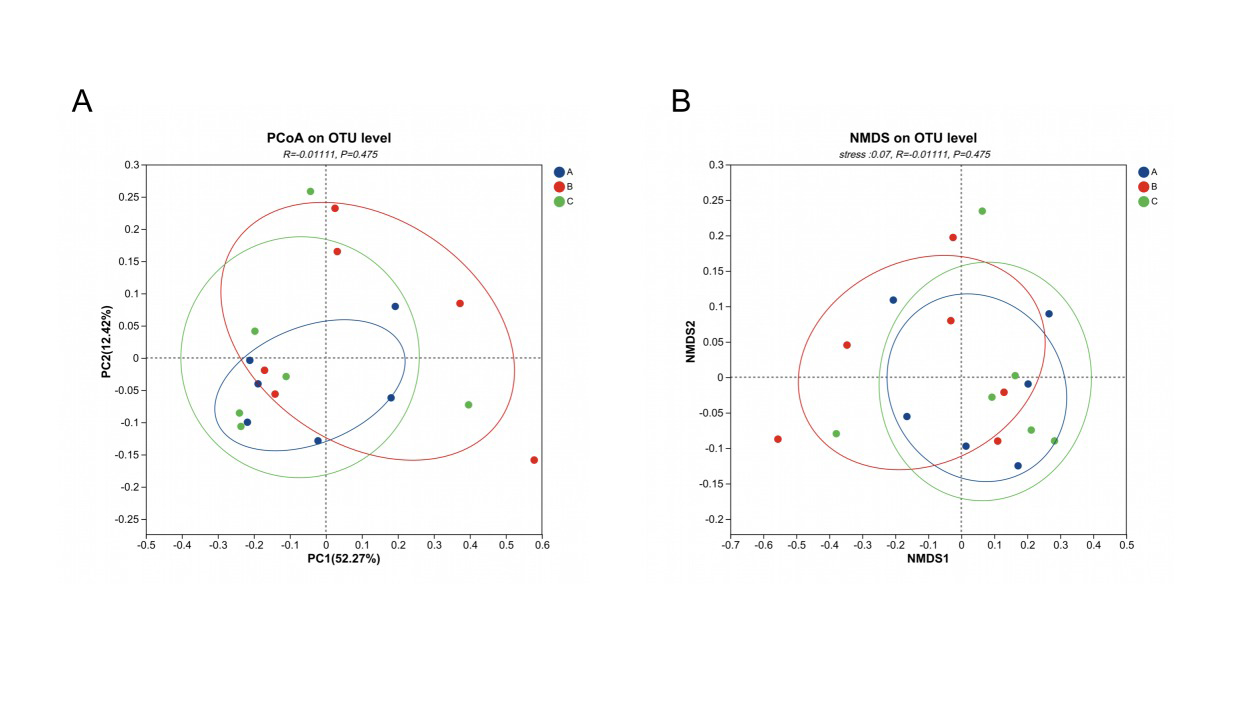


**Fig. S13** Effects of dietary supplementation of rumen-protected and unprotected glucose on the β-diversity of jejunum microorganisms in Xinjiang Brown cattle. **A** PCoaA on OTU level. **B** NMDS on OTU level.


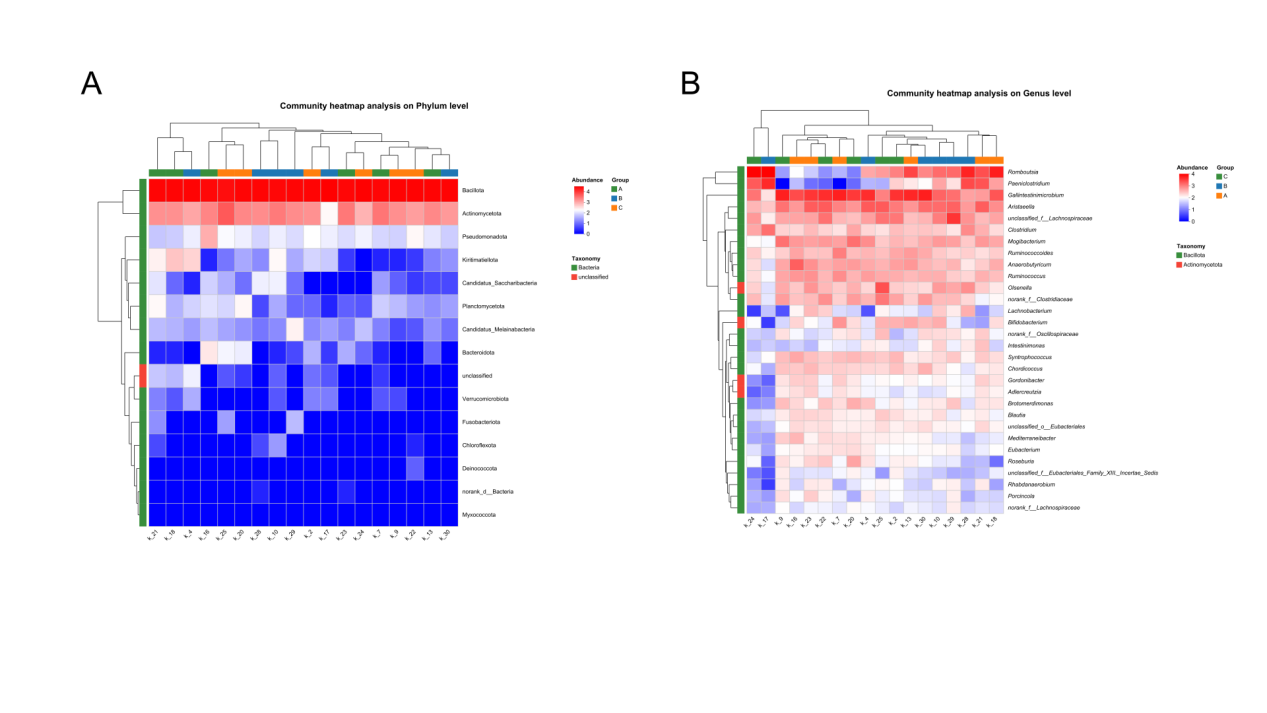


**Fig. S14** The differences in the relative abundance of bacteria between treatment groups in jejunum fluid samples of Xinjiang Brown cattle. **A** Relative abundances of the top 15 bacterial taxa at the phylum level. **B** Relative abundances of the top 30 bacterial taxa at the genus level.


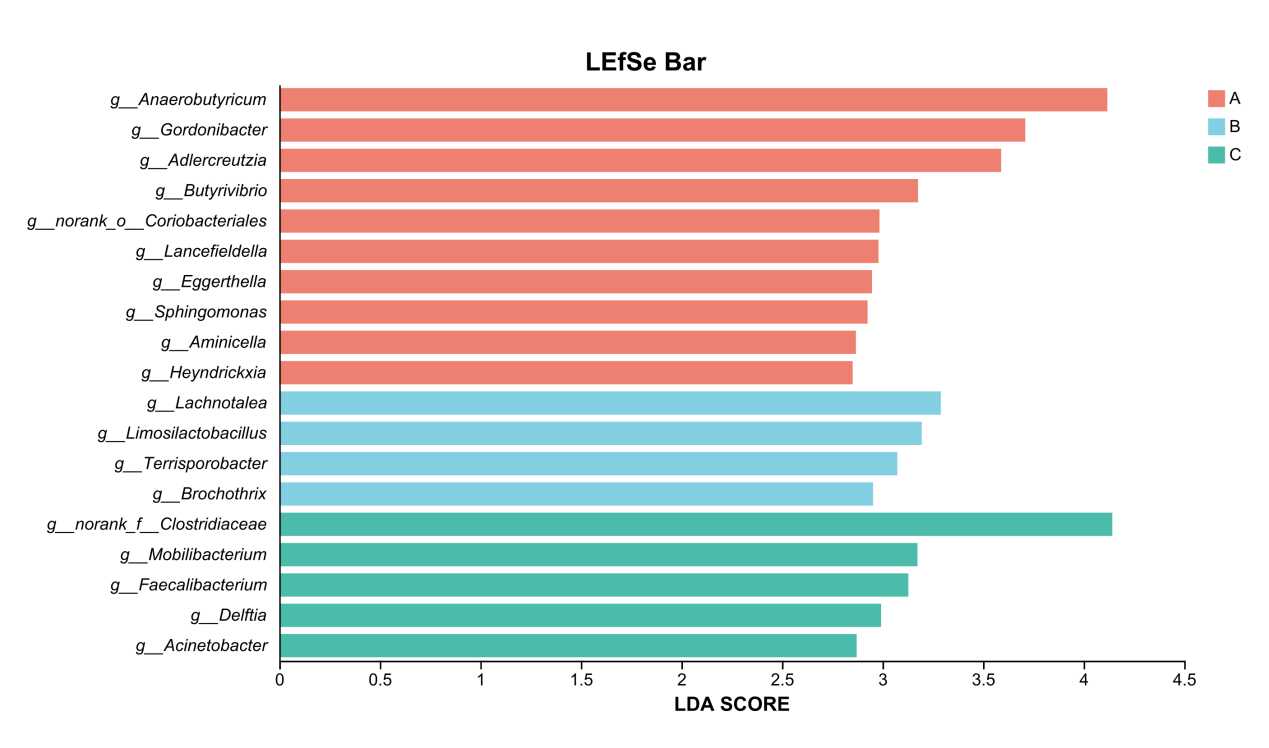


**Fig. S15** The significantly differential microorganisms based on the linear discriminant analysis effect size (LEfSe) cladogra in jejunum fluid samples of Xinjiang Brown cattle among treatment groups.


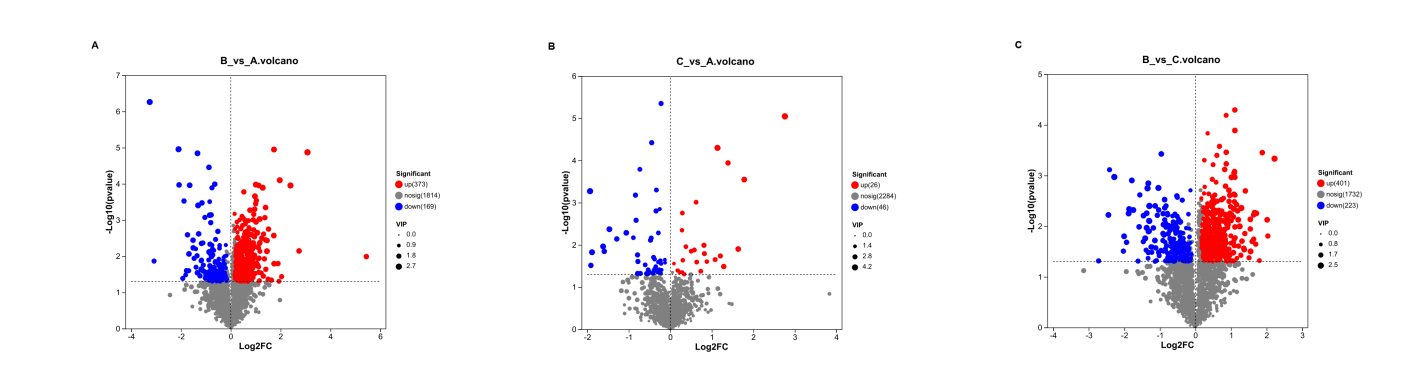


**Fig. S16** The difference of metabolites in jejunum fluid samples of Xinjiang Brown cattle among treatment groups. **A** The amount of data of up-regulated and down-regulated differential metabolites between group A and group B. **B** The amount of data of up-regulated and down-regulated differential metabolites between group B and group C. **C** The amount of data of up-regulated and down-regulated differential metabolites between group A and group C.


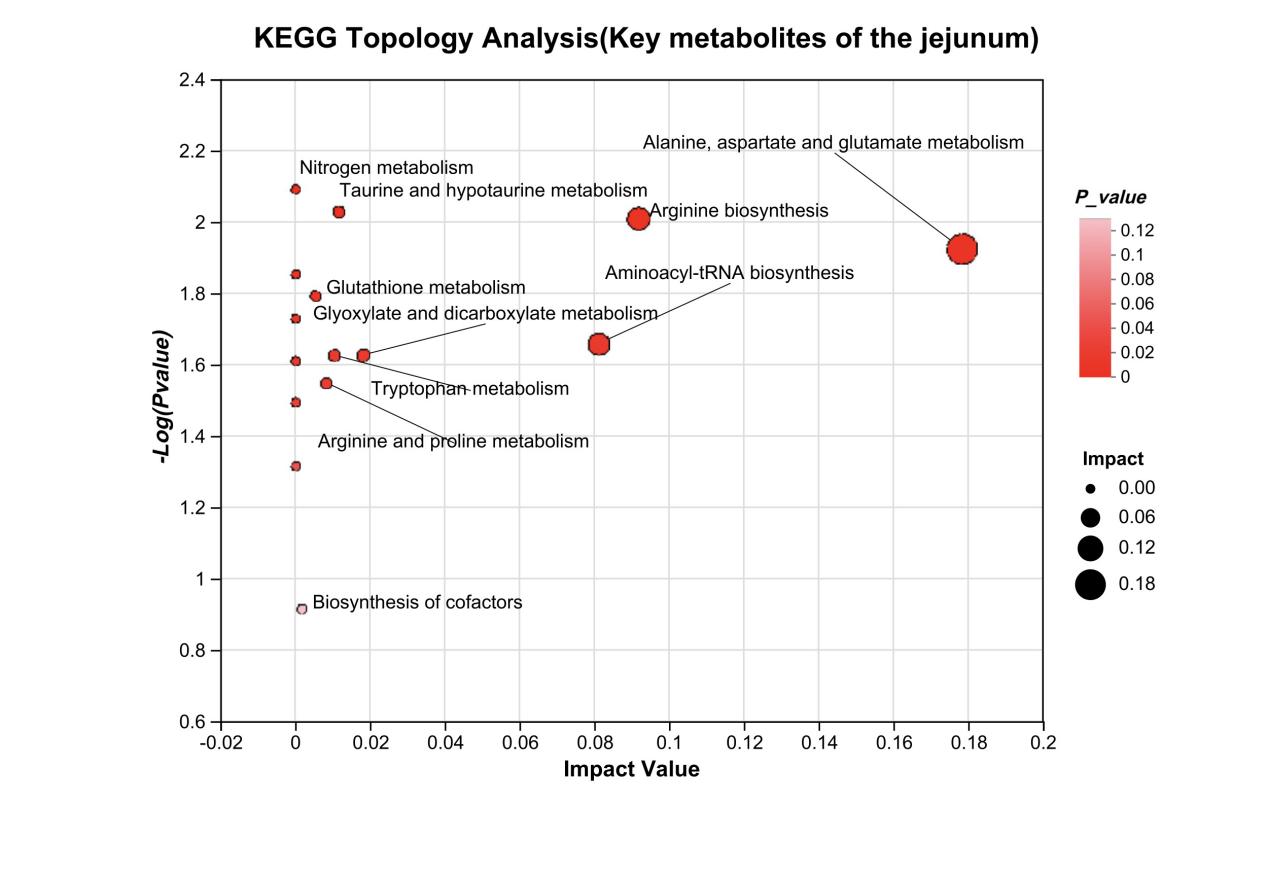


Fig. S17 The differential pathways enriched by Key metabolites (17) ensembles in jejunum fluid samples of Xinjiang Brown cattle.
